# Relationship of SARS-CoV-2–specific CD4 response to COVID-19 severity and impact of HIV-1 and tuberculosis coinfection

**DOI:** 10.1172/JCI149125

**Published:** 2021-06-15

**Authors:** Catherine Riou, Elsa du Bruyn, Cari Stek, Remy Daroowala, Rene T. Goliath, Fatima Abrahams, Qonita Said-Hartley, Brian W. Allwood, Nei-Yuan Hsiao, Katalin A. Wilkinson, Cecilia S. Lindestam Arlehamn, Alessandro Sette, Sean Wasserman, Robert J. Wilkinson, on behalf of the HIATUS consortium

**Affiliations:** 1 Wellcome Centre for Infectious Disease Research in Africa and Institute of Infectious Disease and Molecular Medicine,; 2 Division of Medical Virology, Department of Pathology, and; 3 Department of Medicine, University of Cape Town, Cape Town, South Africa.; 4 Department of Infectious Diseases, Imperial College London, London, United Kingdom.; 5 Department of Radiology, University of Cape Town, Cape Town, South Africa.; 6 Division of Pulmonology, Department of Medicine, Stellenbosch University and Tygerberg Hospital, Cape Town, South Africa.; 7 National Health Laboratory Service, University of Cape Town and Groote Schuur Hospital, Cape Town, South Africa.; 8 The Francis Crick Institute, London, United Kingdom.; 9 Center for Infectious Disease, La Jolla Institute for Immunology, La Jolla, California, USA.; 10 Department of Medicine, School of Medicine, University of California San Diego, La Jolla, California, USA.; 11 Members of the HIATUS consortium are detailed in Supplemental Acknowledgments.

**Keywords:** AIDS/HIV, COVID-19, Cellular immune response, T cells, Tuberculosis

## Abstract

T cells are involved in control of coronavirus disease 2019 (COVID-19), but limited knowledge is available on the relationship between antigen-specific T cell response and disease severity. Here, we used flow cytometry to assess the magnitude, function, and phenotype of SARS coronavirus 2–specific (SARS-CoV-2–specific) CD4^+^ T cells in 95 hospitalized COVID-19 patients, 38 of them being HIV-1 and/or tuberculosis (TB) coinfected, and 38 non–COVID-19 patients. We showed that SARS-CoV-2–specific CD4^+^ T cell attributes, rather than magnitude, were associated with disease severity, with severe disease being characterized by poor polyfunctional potential, reduced proliferation capacity, and enhanced HLA-DR expression. Moreover, HIV-1 and TB coinfection skewed the SARS-CoV-2 T cell response. HIV-1–mediated CD4^+^ T cell depletion associated with suboptimal T cell and humoral immune responses to SARS-CoV-2, and a decrease in the polyfunctional capacity of SARS-CoV-2–specific CD4^+^ T cells was observed in COVID-19 patients with active TB. Our results also revealed that COVID-19 patients displayed reduced frequency of *Mycobacterium tuberculosis*–specific CD4^+^ T cells, with possible implications for TB disease progression. These results corroborate the important role of SARS-CoV-2–specific T cells in COVID-19 pathogenesis and support the concept of altered T cell functions in patients with severe disease.

## Graphical abstract

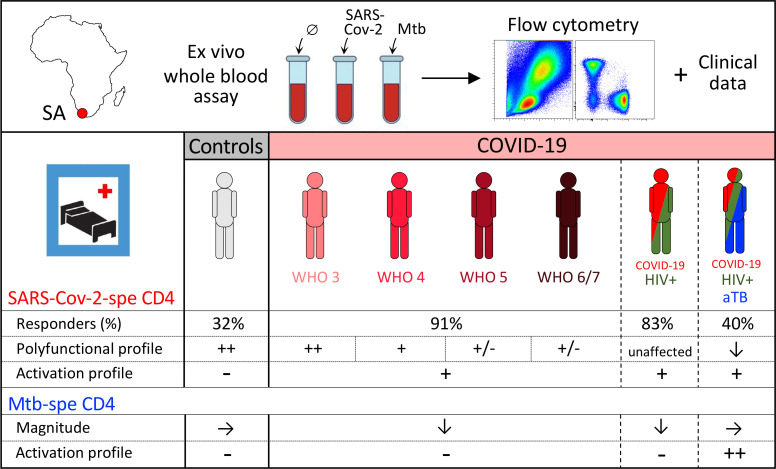

## Introduction

Coronavirus disease 2019 (COVID-19), caused by SARS coronavirus 2 (SARS-CoV-2), emerged in December 2019 and is the cause of a devastating pandemic resulting in more than 100 million infections and over 2 million deaths within the last year. COVID-19 shows an extremely variable clinical course, ranging from an asymptomatic state or mild respiratory symptoms to severe viral pneumonia with or without acute respiratory distress syndrome (1). Although most COVID-19 cases are mild (~80%), up to a quarter of people infected with SARS-CoV-2 present with severe disease necessitating hospitalization, and approximately 5% of critical cases require intensive care, putting extreme pressure on health systems. Severe disease is most commonly observed in males, older people, and individuals with preexisting comorbidities (such as hypertension, type 2 diabetes, obesity, or chronic lung disease; ref. 2). Immunologically, COVID-19 severity has been associated with major systemic alterations of the host immune system, including profound lymphopenia, skewed distribution and activation of T cell subpopulations, disruption of the B cell compartment, and elevated plasma concentrations of proinflammatory cytokines (3–7). A growing body of evidence suggests that SARS-CoV-2–specific T cell response plays a key role in modulating COVID-19 pathogenesis (8–13). Although the precise nature of T cell responses conferring protection is still unclear, it is now well established that SARS-CoV-2 elicits a broad T cell response in the majority of patients, with CD4 responses being dominant over CD8 (14). Moreover, preexisting SARS-CoV-2 cross-reactive T cells may also contribute to the divergent manifestations of COVID-19. These cells, likely acquired during previous infections with endemic human coronaviruses, have been identified in 20% to 50% of individuals unexposed to SARS-CoV-2 in different populations around the world (8, 10, 15–20). It is yet to be determined whether preexisting immunity to SARS-CoV-2 is sufficient to confer protection or attenuate the severity of COVID-19. Our knowledge regarding SARS-CoV-2–induced immune responses is rapidly expanding, yet very few studies have simultaneously examined the magnitude or functional and phenotypical profile of SARS-CoV-2–responding T cells in relation to disease severity. This represents an important gap in our understanding of the role played by T cells during the clinical course of COVID-19, which has implications for pathogenesis and the assessment of vaccine efficacy.

Importantly, in countries with a high burden of HIV-1 and *Mycobacterium tuberculosis* (*M. tuberculosis*) infections, the intersecting coronavirus, HIV-1, and TB epidemics pose additional public health challenges. HIV infection induces a profound dysregulation of both the innate and adaptive immune systems (21), weakening the host’s ability to mount and/or maintain immune responses to other pathogens or upon vaccination (22, 23). Furthermore, immune dysfunctions often persist despite antiretroviral therapy (ART; ref. 24). It is therefore likely that HIV-1 infection will impair the SARS-CoV-2 immune response. Likewise, coinfection with active tuberculosis (aTB) and COVID-19 is also of particular concern. Both diseases are primarily respiratory illnesses, eliciting a hyperinflammatory state in the lung. It is thus reasonable to speculate that the hyperinflammatory milieu induced by COVID-19 could accelerate TB disease progression and vice versa (25, 26). Moreover, profound lymphopenia and hyperinflammation associated with COVID-19 could favor *M. tuberculosis* reactivation. These concerns are further underlined by several large epidemiological studies showing that HIV-1 and active TB are independently associated with an increased risk of COVID-19–related death (27–33). It is thus an urgent research priority to investigate the profile of the SARS-CoV-2–specific T cells in patients coinfected with HIV-1 and/or active TB and to assess the impact of acute SARS-CoV-2 infection on the *M. tuberculosis*-specific memory CD4^+^ T cell response.

In this study, focused on pathogen-specific CD4^+^ T cell responses, our aims were to (a) compare the profile of preexisting SARS-CoV-2 cross-reactive CD4^+^ T cells and COVID-19–induced CD4^+^ T cells, (b) define the relationship between COVID-19 severity and the SARS-CoV-2–specific CD4^+^ T cell response, (c) investigate the impact of HIV and/or active TB coinfection on the SARS-CoV-2–specific CD4^+^ T cell response, and (d) assess the effect of COVID-19 on the *M. tuberculosis*–specific CD4^+^ T cell response.

## Results

### Clinical characteristics of the study participants.

Using a whole-blood assay, we investigated the SARS-CoV-2– and *M. tuberculosis*–specific CD4^+^ T cell response in hospitalized COVID-19 (*n* = 95) and non–COVID-19 patients (*n* = 38). The clinical characteristics of patients are presented in Table 1. COVID-19 cases were defined based on a documented positive SARS-CoV-2 PCR swab result; hospitalized non–COVID-19 patients all had a SARS-CoV-2 PCR-negative result and no detectable SARS-CoV-2 nucleocapsid–specific IgG measured in blood collected at enrollment. Samples were collected at a median of 2 days (IQR: 1–4) after admission to the hospital in Cape Town, South Africa. The median age was comparable between the 2 groups (52 vs. 51 years) and males predominated in the COVID-19 group (57.9% vs. 34.2%, *P* = 0.014). A high proportion of comorbidities, such as hypertension (46.3%), diabetes (32.6%), and obesity (26.3%), was reported in the COVID-19 group. The non–COVID-19 controls were well-matched to the COVID-19 group in terms of prevalence of hypertension, diabetes, and obesity. However, a greater proportion of non–COVID-19 controls had cardiovascular disease (44.7% vs. 7.3%, *P* *<* 0.0001) and other respiratory diseases, including asthma, chronic obstructive pulmonary disease, or bronchiectasis (28.9% vs. 2.1%, *P* < 0.0001), compared with the COVID-19 group. COVID-19 patients had a range of different requirements for oxygen therapy and supportive care, as reflected by their World Health Organization (WHO) ordinal scale score (see Methods), with approximately half being classified as mild/moderate cases (WHO < 5) and the other half as severe cases (WHO 5 or higher; ref. 34). Most non–COVID-19 controls did not require oxygen therapy (57.9%). The majority of the COVID-19 patients received treatment with steroids (78.9%) following the outcome of the RECOVERY trial (35).

About 1/3 of the recruited participants were HIV-1 infected (*n* = 31). In the COVID-19 group, the majority of HIV-1–infected patients were on ART (74.2%) and had a median CD4 count of 132 cells/mm^3^ and a median log viral load less than 1.3 log mRNA copies/mL. HIV-1–infected non–COVID-19 controls had a lower median CD4 count (20 cells/mm^3^, *P* = 0.03) and higher viral loads (5.37 log mRNA copies/mL, *P* = 0.0005) owing to proportionally fewer participants being on ART in this group (46.1%). Last, 15 participants in the COVID-19 group had active TB (8 of them also being HIV-1 infected), and 5 non–COVID-19 controls had active TB (all of them being HIV-1 infected). It is important to mention that most of the HIV-1–infected participants without active TB were virally suppressed (77.3%, 17/22), whereas only 1 of the 7 HIV-1–infected participants with active TB was aviremic. Further details on the clinical characteristics of the HIV-infected group and the HIV/aTB-coinfected group are presented in Supplemental Table 1; supplemental material available online with this article; https://doi.org/10.1172/JCI149125DS1

The comparisons of the clinical characteristics between discharged and deceased patients are presented in Supplemental Table 2; 29.5% (28/95) COVID-19 patients died. As previously reported, COVID-19 patients who died were older, were predominantly male, had more severe disease according to their WHO ordinal scale classification, and were characterized by elevated systemic inflammation. No deaths occurred in the non–COVID-19 control group.

### Measures of COVID-19 severity.

**Figure 1:**
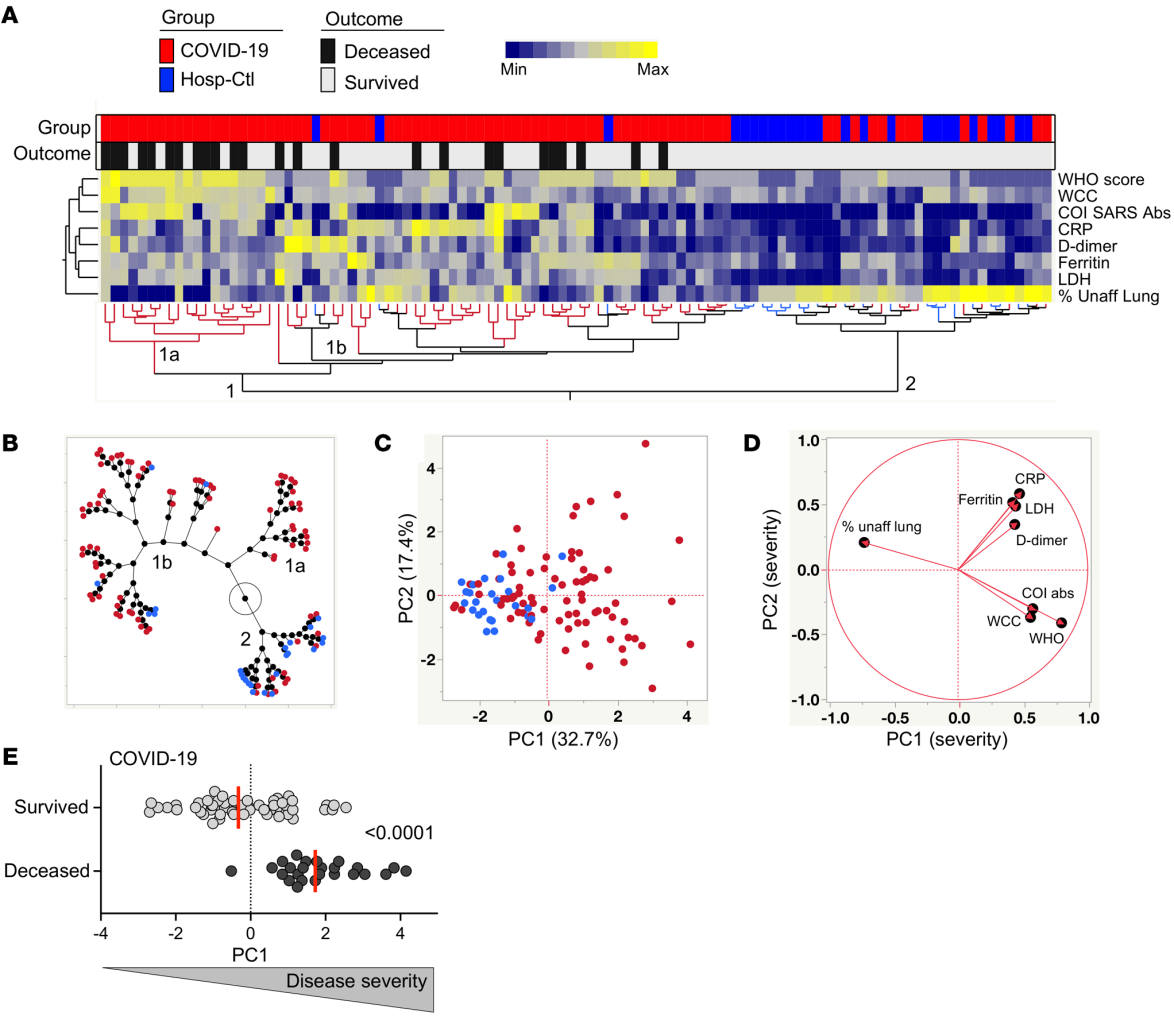
Measures of COVID-19 disease severity. (**A**) An unsupervised 2-way hierarchical cluster analysis (HCA, Ward’s method) was employed to grade COVID-19 disease, using the WHO ordinal scale scoring, Roche Elecsys anti–SARS-CoV-2 antibody cutoff index, WCC, CRP, D-dimer, ferritin, LDH, and radiographic evidence of disease extent expressed as percentage of unaffected lung. COVID-19 status (COVID-19 cases in red and SARS-CoV-2–uninfected hospitalized controls in blue) and outcome (survived in white and deceased in black) of each patient is indicated at the top of the dendrogram. Data are depicted as a heatmap colored from minimum to maximum values detected for each parameter. (**B**) Constellation plot-cluster analysis based on all measured parameters. Each dot represents a participant and is color-coded according to his or her COVID-19 status. Each cluster obtained for the HCA is identified by a number. (**C**) Principal component analysis (PCA) on correlations, based on the 8 clinical parameters, was used to explain the variance of the data distribution in the cohort. Each dot represents a participant. The 2 axes represent principal components 1 (PC1) and 2 (PC2). Their contribution to the total data variance is shown as a percentage. (**D**) Loading plot showing how each parameter influences PC1 and PC2 values. (**E**) Comparison of PC1 score values between COVID-19 cases who survived and those who died. Bars represent medians. Statistical comparisons were calculated using the nonparametric Mann-Whitney *U* test. Only participants with complete clinical data were included in the analysis (*n* = 79 COVID-19 patients and *n* = 25 hospitalized controls).

The WHO ordinal scale, stratifying patients according to their oxygen therapy requirement, has been widely used as a correlate of COVID-19 severity. Additionally, a wide range of nonspecific indicators of systemic inflammation, including among others C-reactive protein (CRP), ferritin, serum amyloid A (SAA), procalcitonin, lactate dehydrogenase (LDH), D-dimer, IL-6, IL-10, white cell count (WCC), or neutrophil count, have been associated with adverse COVID-19 outcomes (36–39). Furthermore, higher levels of SARS-CoV-2–specific antibodies have been shown to associate with increased COVID-19 severity (40, 41). In this study cohort, we also observed an increased level of SARS-CoV-2–specific antibodies in patients with severe COVID-19 defined by the WHO ordinal scale (Supplemental Figure 1). Thus, based on the clinical data available in this study, 8 clinical parameters were combined to perform a hierarchical clustering analysis, including WHO ordinal scale scoring, Roche Elecsys anti–SARS-CoV-2 antibody cutoff index, WCC, CRP, D-dimer, ferritin, LDH, and radiographic evidence of disease expressed as the percentage of unaffected lung. Two main clusters were identified: cluster 1 encompassed almost exclusively COVID-19 cases (92%), and cluster 2 contained 62.5% of hospitalized SARS-CoV-2–uninfected controls and 37.5% of COVID-19 cases. Moreover, 2 subgroups emerged from cluster 1, where cluster 1a was enriched in COVID-19 patients who died (Figure 1, A and B). Principal component analysis (PCA) showed a good separation between COVID-19 cases and hospitalized non–COVID-19 controls in which PC1 accounted for 32.7% and PC2 17.4% of the variance (Figure 1C). The corresponding loading plot shows that the lung percentage unaffected score, oxygen therapy requirement, and WCC were the main drivers of PC1 variance (Figure 1D). Furthermore, the PC1 score in COVID-19 patients who died was significantly higher (*P* < 0.0001) compared with patients who survived (Figure 1E). This analytical approach grades disease severity as a continuum, allowing the simultaneous integration of multiple clinical parameters of known relevance in COVID-19 outcome.

### Distinct phenotype of SARS-CoV-2–responding CD4^+^ T cells in COVID-19 and non–COVID-19 patients.

**Figure 2:**
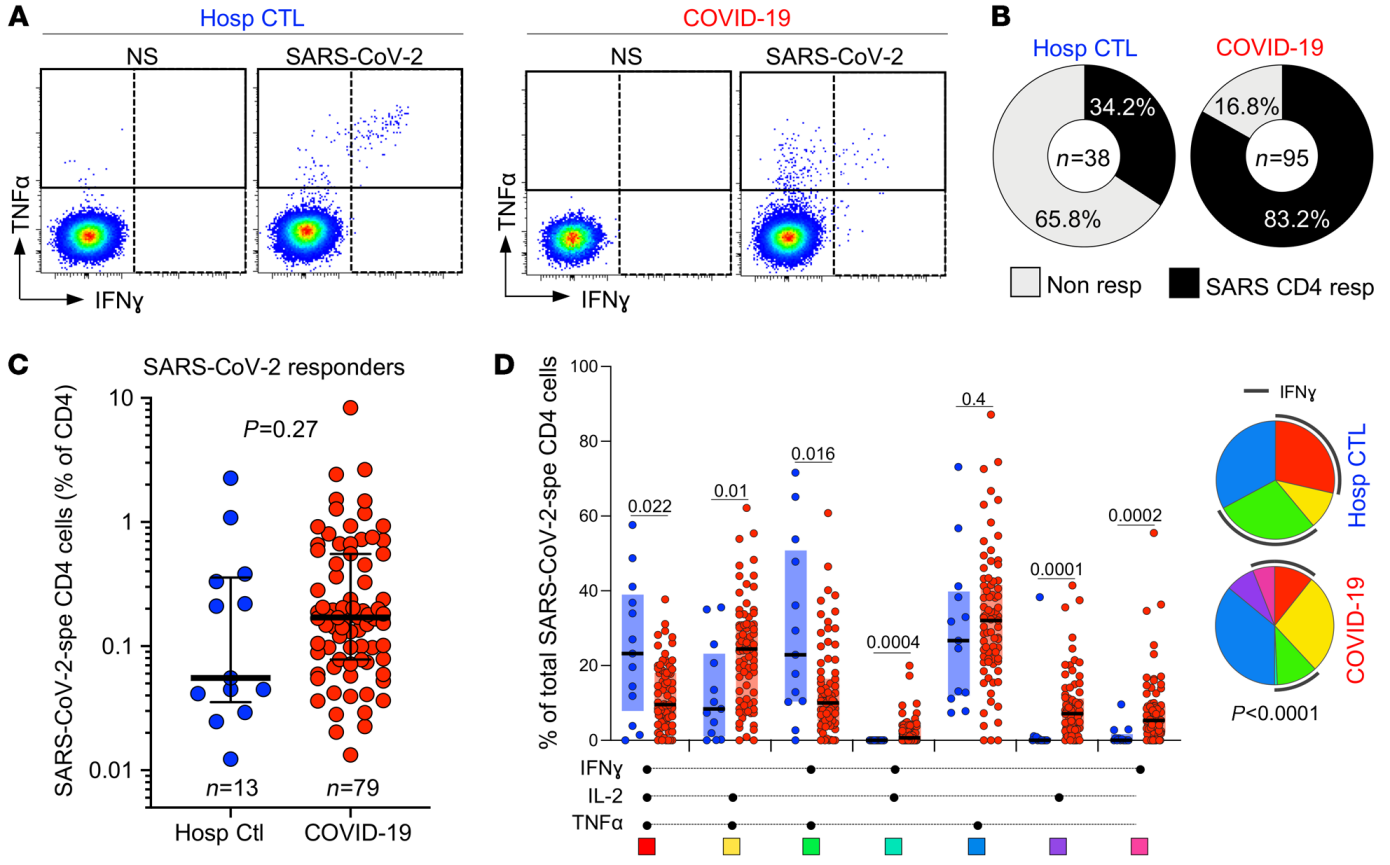
Prevalence, magnitude, and functional profile of SARS-CoV-2–specific CD4^+^ T cells between COVID-19 cases and SARS-CoV-2–uninfected hospitalized patients. (**A**) Representative flow cytometry plots of IFN-γ and TNF-α expression. NS, no stimulation. (**B**) Proportion of patients exhibiting a detectable SARS-CoV-2 CD4 response in each group. The number of studied patients is indicated in the pie (*n* = 79 COVID-19 patients and *n* = 25 hospitalized controls). (**C**) Frequency of SARS-CoV-2–specific CD4^+^ T cells in hospitalized control (blue, *n* = 13) and COVID-19 responders (red, *n* = 79). Statistical comparisons were calculated using the nonparametric Mann-Whitney *U* test. (**D**) Polyfunctional profile of SARS-CoV-2–specific CD4^+^ T cells in hospitalized controls and COVID-19 patients. The median and IQR are shown. Each response pattern is color-coded, and data are summarized in the pie charts. Wilcoxon’s rank test was used to compare response patterns between groups. Statistical differences between pies were defined using a permutation test.

First, we compared the prevalence, magnitude, and phenotypical profile of SARS-CoV-2–responding CD4^+^ T cells (e.g., cells producing IFN-γ, TNF-α, or IL-2; Figure 2A) between hospitalized non–COVID-19 controls and confirmed COVID-19 patients. SARS-CoV-2–reactive CD4^+^ T cells were detected in 34.2% (13/38) of non–COVID-19 controls, whereas 83.2% (79/95) of COVID-19 patients exhibited a SARS-CoV-2–specific response (Figure 2B). These data concord with several publications demonstrating the presence of preexisting SARS-CoV-2 cross-reactive CD4^+^ T cells in 20% to 50% of SARS-CoV-2–unexposed individuals (8, 10, 15–18). We observed high variability in the magnitude of the SARS-CoV-2 CD4^+^ T cell response among the SARS-CoV-2–responding participants from both groups; although not statistically significant, the median response in COVID-19 cases was approximately 3-fold higher compared with non–COVID-19 controls (0.17%, IQR: 0.08%–0.55% and 0.05%, IQR: 0.03%–0.36%, respectively; Figure 2C). Of note, in the COVID-19 group, the frequency of SARS-CoV-2–specific CD4^+^ T cells strongly associated with the magnitude of SARS-CoV-2 nucleocapsid–specific IgG (*P* < 0.0001, *r* = 0.61; Supplemental Figure 2A), as previously reported (8, 15, 42, 43).

When cytokine responses were analyzed individually, TNF-α was the predominant cytokine produced by CD4 cells in response to SARS-CoV-2 peptides. A short-term (5-hour) whole-blood assay in both groups revealed that TNF-α production was significantly higher compared with IL-2 and IFN-γ (Supplemental Figure 2B). Combined analyses of all measured cytokines (IL-2, IFN-γ, and TNF-α) showed that the overall polyfunctional profile of SARS-CoV-2–specific cells in COVID-19 participants was distinct from uninfected controls (*P* < 0.0001). In COVID-19, the CD4 response was characterized by limited expression of IFN-γ and was enriched in cells coexpressing IL-2 and TNF-α. Conversely, in non–COVID-19 controls, most SARS-CoV-2–reactive CD4^+^ T cells were distributed between triple functional cells (IL-2^+^IFN-γ^+^TNF-α^+^) and cells coproducing IFN-γ and TNF-α (Figure 2D).

**Figure 3:**
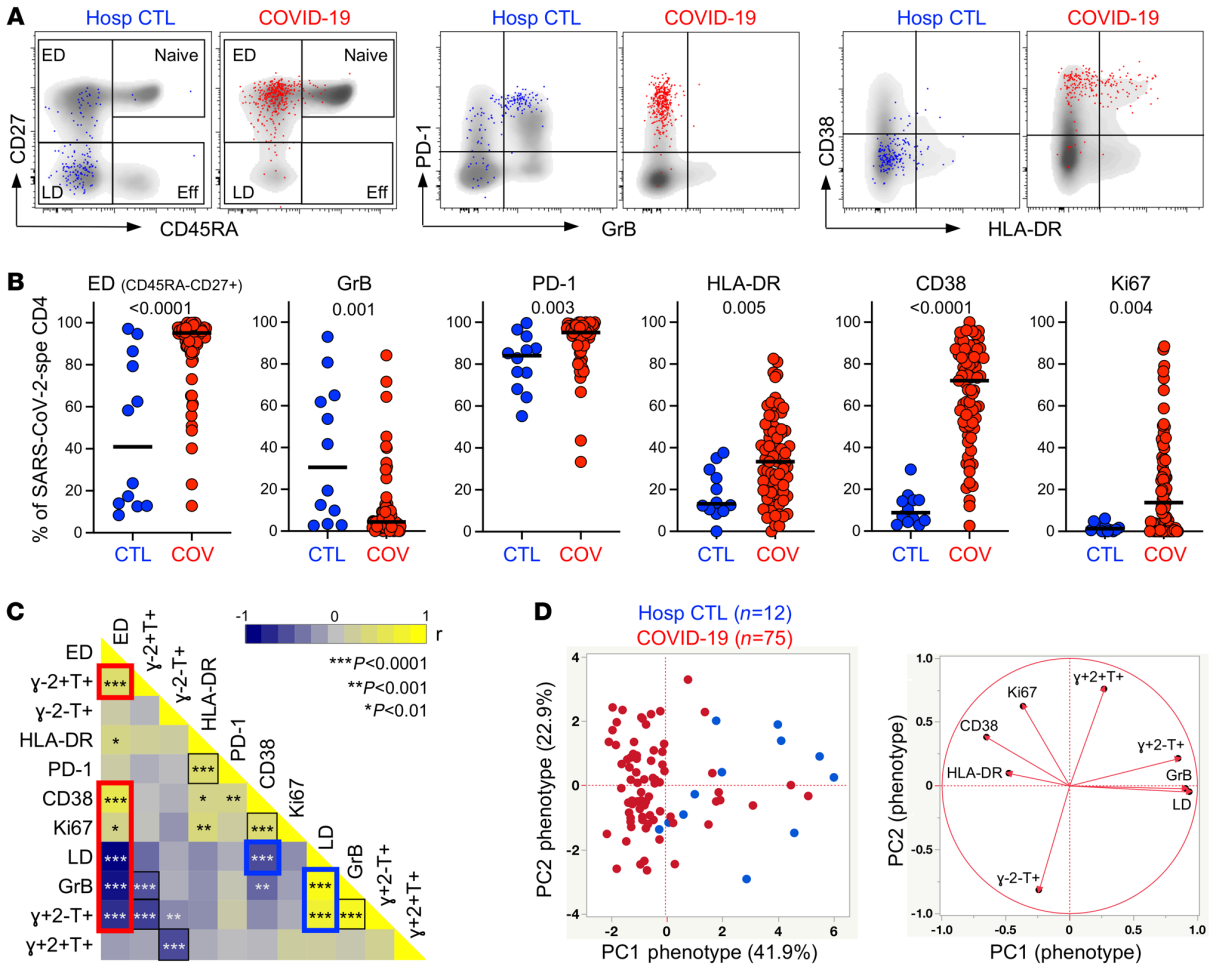
Memory and activation profile of SARS-CoV-2–specific CD4^+^ T cells between COVID-19 cases and SARS-CoV-2–uninfected hospitalized patients. (**A**) Overlay flow plots of CD45RA, CD27, PD-1, GrB, CD38, and HLA-DR expression. Dots depict SARS-CoV-2–specific CD4^+^ T cells and density plots depict total CD4^+^ T cells. Four memory subsets can be delineated: naive (CD45RA^+^CD27^+^), early differentiated (ED, CD45RA^–^CD27^+^), late differentiated (LD, CD45RA^–^CD27^–^), and effector (Eff, CD45RA^+^CD27^–^). (**B**) Summary graphs of the expression of each marker in SARS-CoV-2–specific CD4^+^ T cells (*n* = 75 COVID-19 patients and *n* = 12 hospitalized controls). The phenotype of SARS-CoV-2–specific CD4^+^ T cells was assessed only in those with response greater than 20 events. Bars represent medians. Statistical comparisons were calculated using the nonparametric Mann-Whitney *U* test. (**C**) Heatmap of pairwise Spearman’s correlations between phenotypical and functional traits of SARS-CoV-2–specific CD4^+^ T cells. Spearman’s rank *r* correlation values are shown from blue, –1, to yellow, 1. The red box identifies the profile of ED SARS-CoV-2–specific CD4^+^ T cells and the blue box the profile of LD cells enriched in hospitalized controls. (**D**) PCA (left) based on the 8 phenotypical and functional attributes of SARS-CoV-2–specific CD4^+^ T cells (LD, GrB, HLA-DR, Ki67, CD38 and the proportion of IFN-γ^+^IL-2^+^TNF-α^+^, IFN-γ^+^IL-2^–^TNF-α^+^, and IFN-γ^–^IL-2^–^TNF-α^+^ cells) and corresponding loading plot (right).

We next assessed the memory differentiation (CD27, CD45RA), cytotoxic potential (granzyme B [GrB]), and activation profile (HLA-DR, CD38, Ki67, programmed cell death protein 1 [PD-1]) of SARS-CoV-2–responding CD4^+^ T cells (Figure 3A). In COVID-19 patients, SARS-CoV-2–specific CD4^+^ T cells almost exclusively displayed an early differentiated memory phenotype (CD45RA^–^CD27^+^, median: 95.1%, IQR: 88.7%–97.4%). By contrast, in non–COVID-19 controls, the memory profile of SARS-CoV-2–reactive CD4^+^ T cells was highly variable between individuals, with 50% exhibiting predominantly a late differentiation profile (CD45RA^–^CD27^–^). Moreover, the SARS-CoV-2 response in uninfected controls was characterized by significantly elevated expression of GrB compared with COVID-19 cases (median: 30.6%, IQR: 5%–64.2% vs. 4.4%, IQR: 1.9%–9.6%, respectively, *P* = 0.001).

As expected, the expression of HLA-DR, CD38, and Ki67 on SARS-CoV-2–responding CD4^+^ T cells was significantly higher in COVID-19 cases compared with non–COVID-19 controls (*P* = 0.005, *P* < 0.0001, and *P* = 0.004, respectively), likely reflecting ongoing viral replication (Figure 3B). The expression of CD38 and Ki67 was inversely associated with the time COVID-19 patients spent in clinical care (*P* = 0.0006, *r* = –0.39; and *P* = 0.017, *r* = –0.27, respectively, data not shown). As previously reported (14, 16, 44), in convalescent COVID-19 patients (*n* = 9), although the expression of HLA-DR, CD38, and Ki67 in SARS-CoV-2 CD4^+^ T cells was significantly reduced compared with acute COVID-19 patients (reflecting viral clearance), cells maintained an elevated PD-1 expression and retained their early differentiated phenotype (Supplemental Figure 2C).

Pairwise associations of the functional and phenotypical characteristics of SARS-CoV-2–responding CD4 cells identified 2 signatures: (a) activated cells exhibiting an early differentiated memory phenotype and preferentially secreting IL-2 and TNF-α, characteristic of COVID-19 patients, and (b) late differentiated memory cells with elevated GrB expression endowed with polyfunctional capacities predominantly observed in SARS-CoV-2–responsive CD4^+^ T cells from uninfected individuals (Figure 3C). To determine whether the overall phenotypical profile of SARS-CoV-2–responding CD4^+^ T cells allows discrimination between COVID-19–induced and preexisting cross-reactive CD4 responses, we performed a PCA (Figure 3D) and hierarchical clustering analysis (Supplemental Figure 2D), including 8 parameters (e.g., the proportion of IFN-γ^+^TNF-α^+^IL-2^+^, IFN-γ^–^TNF-α^+^IL-2^+^ IFN-γ^–^TNF-α^+^IL-2^–^ cells; the proportion of the early differentiated memory phenotype; and GrB, HLA-DR, CD38, and Ki67 expression). Both analyses showed that based on the functional and phenotypical traits of SARS-CoV-2–responding CD4^+^ T cells, COVID-19 patients could be distinguished from non–COVID-19 controls.

### The functional and phenotypical signature of SARS-CoV-2–specific CD4^+^ T cells is associated with disease severity.

**Figure 4:**
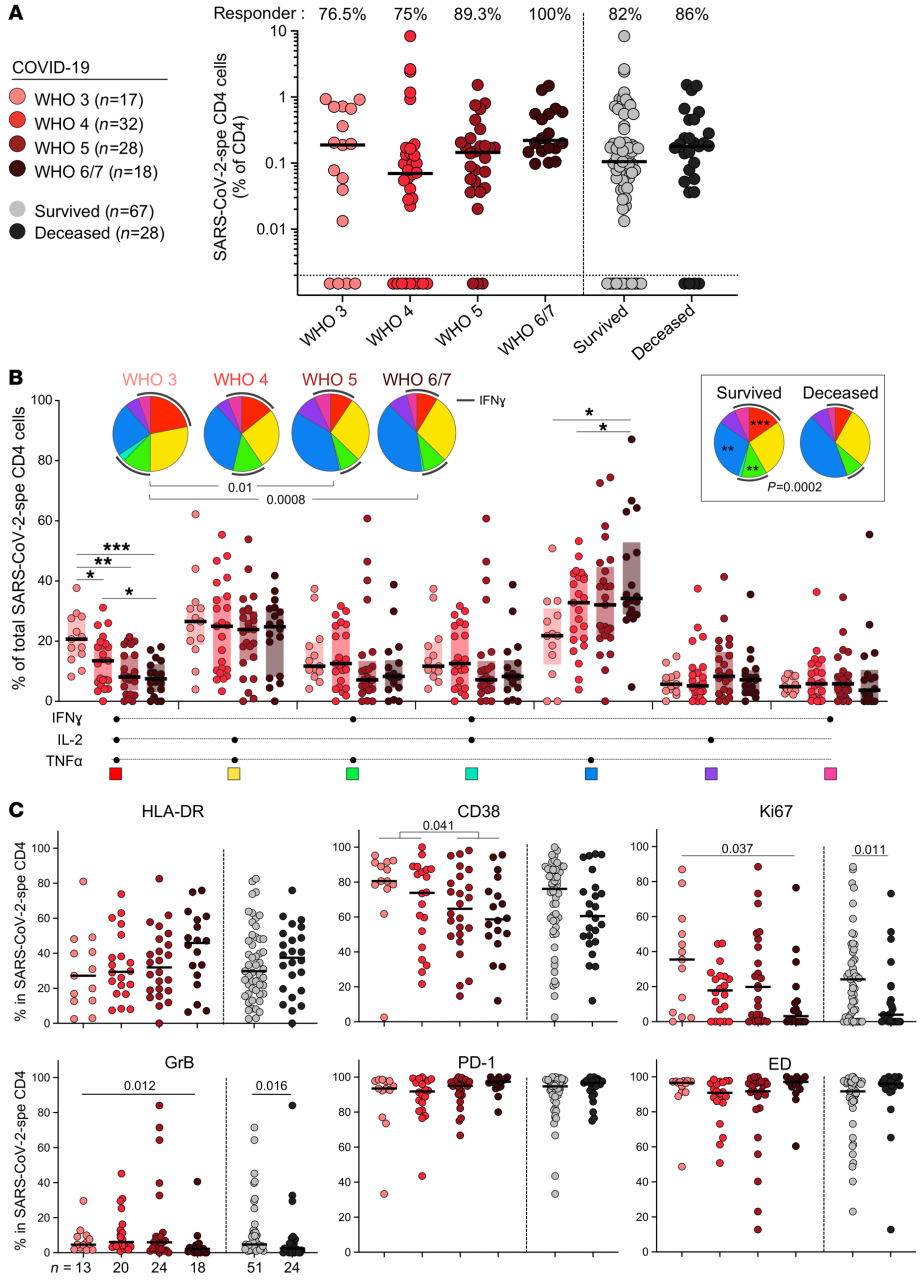
SARS-CoV-2–specific CD4^+^ T cell response in COVID-19 cases stratified by WHO ordinal scale score and outcome. (**A**) Prevalence and frequency of SARS-CoV-2–specific CD4^+^ T cells in COVID-19 cases. Patients were stratified according to WHO ordinal score and outcome. (**B**) Polyfunctional profile of SARS-CoV-2–specific CD4^+^ T cells in COVID-19 cases stratified by WHO score and outcome. Wilcoxon’s rank test was used to compare response patterns between groups (*P < 0.05, **P < 0.01, ***P < 0.001). Statistical differences between pie charts were defined using a permutation test. (**C**) Memory and activation profile of SARS-CoV-2–specific CD4^+^ T cells in COVID-19 cases stratified by WHO score and outcome. The phenotype of SARS-CoV-2–specific CD4^+^ T cells was assessed only in those with response greater than 20 events (*n* = 75 COVID-19 patients). Statistical comparisons were defined using a Kruskal-Wallis test, adjusted for multiple comparisons (Dunn’s test) for the different WHO groups and the Mann-Whitney *U* test to compare COVID-19 patients who survived or died.

We next investigated the relationship between the profile of SARS-CoV-2–specific CD4^+^ T cells and COVID-19 severity. Although no difference was observed in the prevalence or magnitude of SARS-CoV-2–specific CD4 responses based on participants’ WHO ordinal scale score or outcome (survived vs. deceased; Figure 4A), their polyfunctional profile was related to disease severity. Less severe forms of disease were associated with enhanced capacity of SARS-CoV-2–specific CD4^+^ T cells to coexpress IFN-γ, TNF-α, and IL-2. By contrast, TNF-α monofunctional cells were more prevalent in patients with more severe disease (Figure 4B). These functional profiles also related to disease outcome (Figure 4B, inset). Assessing the phenotypical profile of SARS-CoV-2–specific CD4^+^ T cells, the following trends were observed in less severe forms of COVID-19 (WHO 4 or lower): increased expression of CD38, Ki67, and GrB and reduced expression of HLA-DR. However, PD-1 expression and the memory maturation profile of SARS-CoV-2–specific CD4^+^ T cells were comparable between COVID-19 patients stratified by their WHO score (Figure 4C). Of note, no difference in the magnitude of SARS-CoV-2–specific CD4^+^ T cell response was observed between COVID-19 patients receiving steroid treatment or not (*P* = 0.12, data not shown).

**Figure 5:**
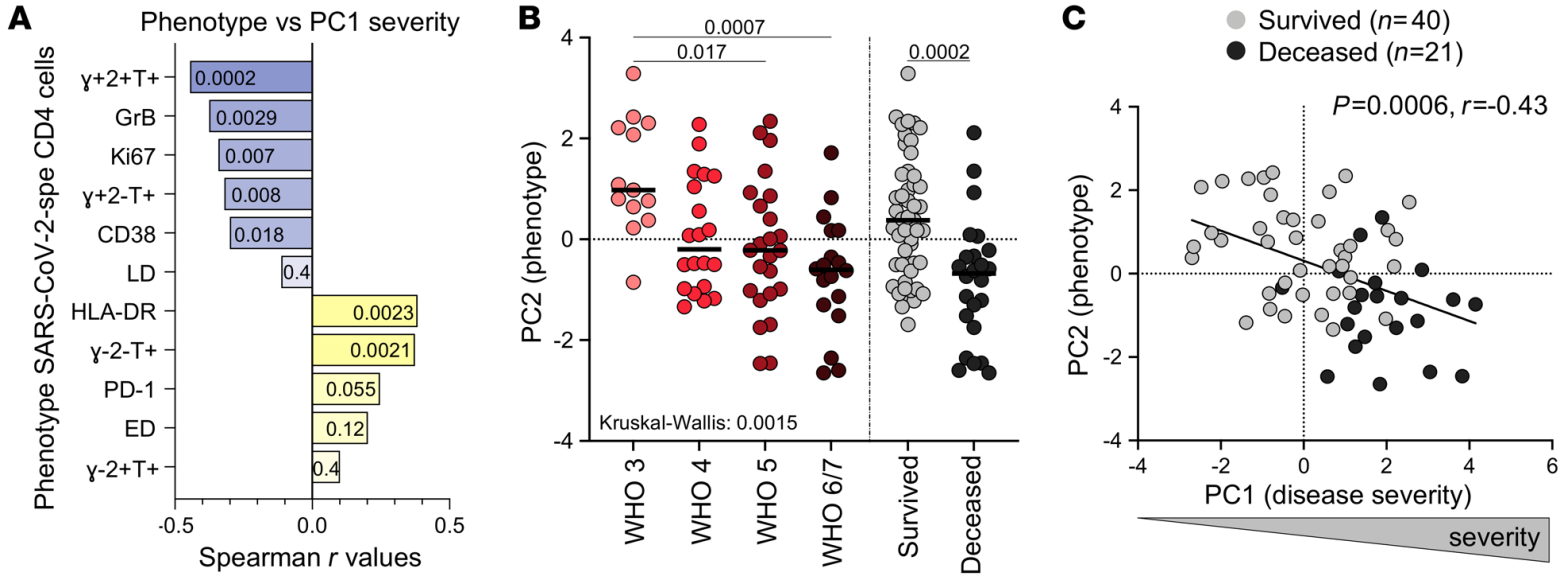
Relationship between COVID-19 severity and functional and phenotypical traits of SARS-CoV-2–specific CD4^+^ T cells. (**A**) Spearman’s correlation *r* values between indicated SARS-CoV-2–specific CD4^+^ T cell features and COVID-19 severity (defined by the composite analysis of clinical parameters, PC1 severity). Negative associations are represented in blue and positive associations in yellow. *P* values are indicated for each comparison. (**B**) Comparison of the overall profile of SARS-CoV-2–specific CD4^+^ T cells (PC2 phenotype) in COVID-19 cases (*n* = 74) stratified by WHO ordinal score and outcome. Statistical comparisons were defined using a Kruskal-Wallis test, adjusted for multiple comparisons (Dunn’s test) for the different WHO groups and the Mann-Whitney *U* test to compare COVID-19 patients who survived or died. (**C**) Association between COVID-19 severity (PC1 severity) and the overall profile of SARS-CoV-2–specific CD4^+^ T cells (PC2 phenotype). COVID-19 survivors are depicted in gray and patients who died in black. Correlation was tested by a 2-tailed nonparametric Spearman’s rank test.

Each functional and phenotypical attribute of SARS-CoV-2–specific CD4^+^ T cells was assessed individually for the strength of its correlation with disease severity (defined by the composite analysis of clinical parameters described in Figure 1C, e.g., PC1 severity). The highest Spearman’s rank *r* values for significant negative correlations were observed between the proportion of IFN-γ^+^IL-2^+^TNF-α^+^ cells, GrB and Ki67 expression, and disease severity; positive associations were found between the proportion of IFN-γ^–^IL-2^–^TNF-α^+^ cells, HLA-DR expression, and disease severity (Figure 5A). Moreover, the global functional and phenotypical pattern of SARS-CoV-2–specific CD4^+^ T cells described in Figure 2H (PC2 phenotype) was associated with patients’ WHO ordinal scale score and outcome (survived vs. deceased; Figure 5B). Overall, COVID-19 severity (PC1 severity) strongly correlated with the traits of SARS-CoV-2–specific CD4^+^ T cells (PC2 phenotype) (*P* = 0.0006, *r* = –0.43, Figure 5C), with severe disease being characterized by poor polyfunctional potential, reduced proliferation capacity, and enhanced HLA-DR expression on SARS-CoV-2–specific CD4^+^ T cells.

### Preexisting lymphopenia impairs the immune response to SARS-CoV-2, and current TB reduces the polyfunctional potential of SARS-CoV-2–specific CD4^+^ T cells.

**Figure 6:**
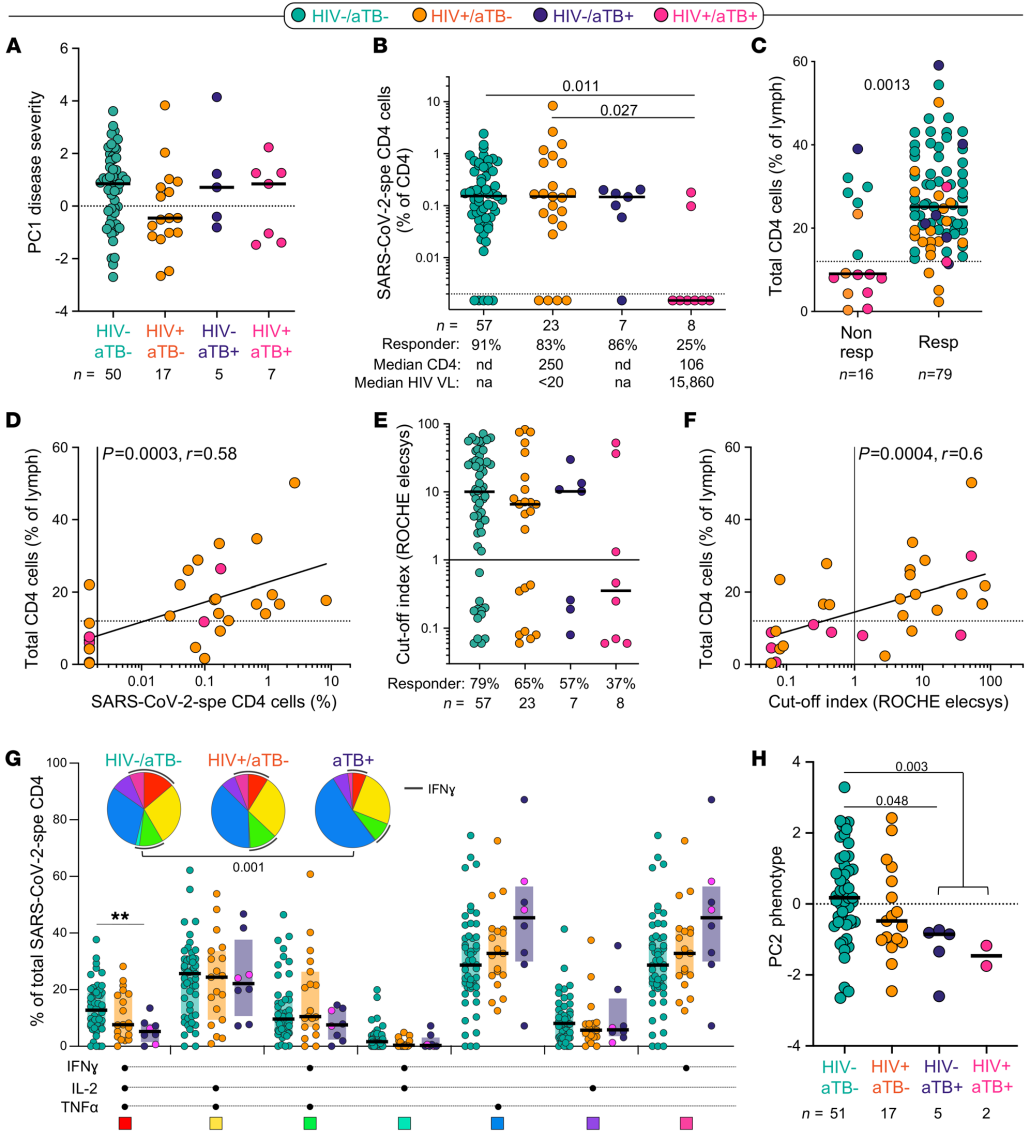
Impact of HIV, aTB, and HIV/aTB coinfection on SARS-CoV-2–specific CD4^+^ T cell response. (**A**) Comparison of COVID-19 severity (defined by the composite analysis of clinical parameters, PC1 severity) between patients grouped according to HIV and/or aTB coinfection. (**B**) Prevalence and frequencies of SARS-CoV-2–specific CD4^+^ T cells in COVID-19 patients stratified by HIV and/or aTB coinfection. Statistical comparisons were defined using a Kruskal-Wallis test adjusted for multiple comparisons (Dunn’s test). (**C**) Comparison of the frequency of total CD4^+^ T cells between SARS-CoV-2 CD4 responders and nonresponders. Dots are color-coded according to patient’s HIV and TB status. Statistical comparison was performed using the Mann-Whitney *U* test. (**D**) Association between the frequency of SARS-CoV-2–specific CD4^+^ T cells and total CD4^+^ T cells in HIV-infected COVID-19 patients. Correlation was tested by a 2-tailed nonparametric Spearman’s rank test. (**E**) Prevalence and magnitude of SARS-CoV-2–specific serological response (defined using the Roche Elecsys assay) in COVID-19 patients stratified by HIV and/or aTB coinfection. (**F**) Association between the magnitude of SARS-CoV-2–specific serological response and the frequency of total CD4^+^ T cells in HIV-infected COVID-19 patients. Correlation was tested by a 2-tailed nonparametric Spearman’s rank test. (**G**) Polyfunctional profile of SARS-CoV-2–specific CD4^+^ T cells in COVID-19 cases stratified by HIV or aTB coinfection. For this analysis, HIV^–^/aTB^+^ and HIV^+^/aTB^+^ patients were combined in 1 group (aTB). Dots are color-coded according to patients’ HIV and TB status. Wilcoxon’s rank test was used to compare response patterns between groups (**P < 0.01). Statistical differences between pie charts were defined using a permutation test. (**H**) Comparison of the overall profile of SARS-CoV-2–specific CD4^+^ T cells (PC2 phenotype) in COVID-19 cases stratified by HIV or aTB coinfection. Statistical comparisons were defined using a Kruskal-Wallis test adjusted for multiple comparisons (Dunn’s test).

Given the systemic inflammation induced by chronic HIV infection and active TB, questions have been raised whether these 2 diseases in particular could distort the immune response to SARS-CoV-2, leading to increased mortality. Indeed, emerging evidence shows that TB and HIV are independently associated with an increased risk for COVID-19 mortality (27, 30, 32). Thus, we defined the impact of HIV, TB, and HIV/aTB coinfection on the magnitude and phenotypical and functional profile of the SARS-CoV-2 CD4^+^ T cell response. Disease severity at enrollment (defined by PC1 severity or WHO ordinal scale on its own) was comparable irrespective of HIV and/or TB coinfection (Figure 6A and data not shown). However, age (an established risk factor for severe disease and mortality), could be a confounder because HIV^+^/aTB^+^, HIV^–^/aTB^+^, and HIV^+^/aTB^–^ patients were significantly younger compared with the HIV^–^/aTB^–^ COVID-19 patients (median: 40, 43, 47, and 55 years, respectively; Supplemental Figure 3A).

Although the proportion of SARS-CoV-2 CD4 responders was similar between HIV^–^/aTB^–^, HIV^+^/aTB^–^, and HIV^–^/aTB^+^ patients (≥83%), in HIV-infected patients with aTB (HIV^+^/aTB^+^), only 25% (2/8) exhibited detectable SARS-CoV-2–specific CD4^+^ T cells (Figure 6B). Of note, among responders, the frequency of SARS-CoV-2–specific CD4^+^ T cells was comparable between all groups (Figure 6B). Given that HIV^+^/aTB^+^ patients are characterized by low absolute CD4 counts (median: 106 cells/mm^3^), we hypothesized that the lack of a SARS-CoV-2–specific response could be related to CD4 lymphopenia. Because recent CD4 count data were not available for all patients, we used the frequency of total CD4^+^ T cells, measured by flow cytometry, as a surrogate measurement of CD4 count: the lowest frequencies of CD4^+^ T cells were observed in participants with HIV-1^+^/aTB^+^ (Supplemental Figure 3B). The frequency of total CD4 cells was significantly higher in SARS-CoV-2 responders compared with nonresponders (median: 25% and 9%, respectively, *P* = 0.0013; Figure 6C). Moreover, in HIV-infected patients, the magnitude of SARS-CoV-2–specific CD4^+^ T cells was associated with the frequency of total CD4^+^ T cells (*P* = 0.0006, *r* = 0.58; Figure 6D) and absolute CD4 count (*P* = 0.001, *r* = 0.59, data not shown). Interestingly, patients coinfected with HIV and aTB also exhibited a limited capacity to generate SARS-CoV-2 antibodies: only 3 out of 8 patients had a positive serology of modest magnitude (Figure 6E). As for the frequency of SARS-CoV-2–specific CD4 response, the magnitude of SARS-CoV-2 antibodies correlated with the frequency of total CD4^+^ T cells in HIV-infected patients (*P* = 0.0011, *r* = 0.56; Figure 6F). Of note, in our cohort, the lack of a SARS-CoV-2–specific CD4 response in patients with aTB was not associated with increased mortality: death was recorded in 4 out of the 8 SARS-CoV-2 CD4 responders and 2 out of the 7 of CD4 nonresponders (data not shown).

We did not observe significant differences in the memory and activation profile of SARS-CoV-2–specific CD4^+^ T cells based on patients’ HIV or TB status (Supplemental Figure 3C). However, in COVID-19 patients with concomitant aTB, SARS-CoV-2–specific CD4^+^ T cells displayed lower polyfunctional capacity, characterized by significant reduction of the cells with 3 functions, compared with HIV^–^/aTB^–^ patients (Figure 6G). Finally, although HIV infection did not significantly alter the functional and phenotypical profile of SARS-CoV-2–specific CD4^+^ T cells, in patients with aTB, the global SARS-CoV-2–specific CD4^+^ T cell pattern was significantly different compared with HIV-uninfected COVID-19 patients (Figure 6H).

### Acute SARS-CoV-2 infection decreases M.

*tuberculosis–specific CD4^+^ T cell response*. Many viruses, including SARS-CoV-2, cause a temporary immunosuppressive effect, which could lead to the reactivation of subclinical bacterial infection (45). Thus, in a TB-endemic country such as South Africa, many concerns have been raised about the possibility that COVID-19 could reactivate latent TB.

**Figure 7:**
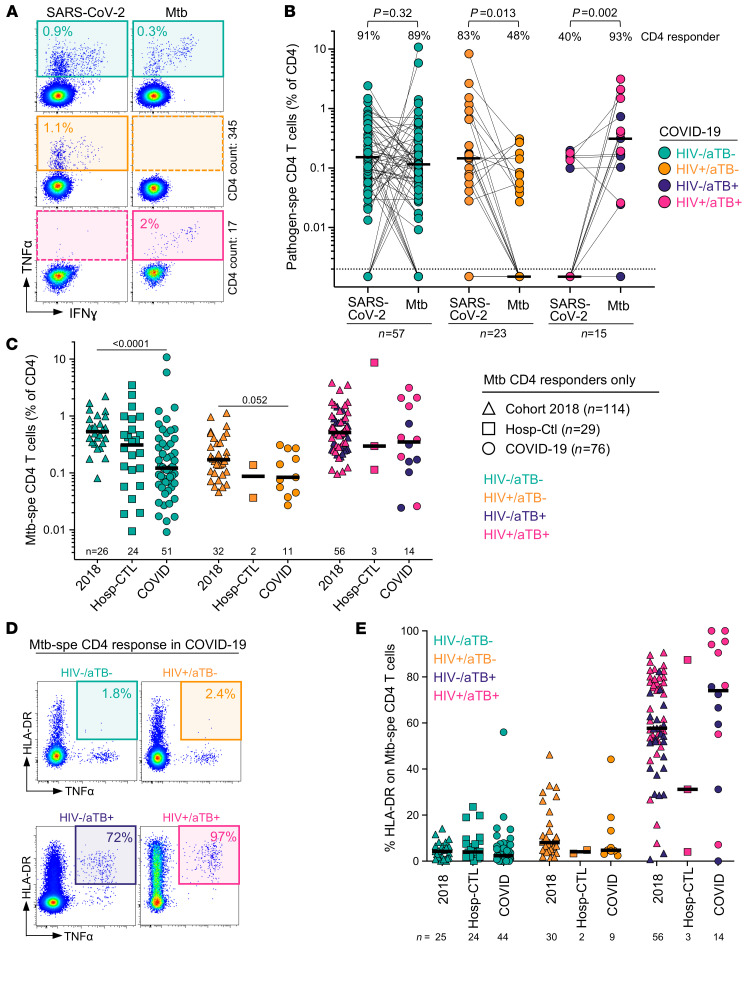
Impact of COVID-19 on *M.* ***tuberculosis*–specific CD4^+^ T cell response.** (**A**) Representative examples of flow cytometry plots of SARS-CoV-2– and *M. tuberculosis*–specific CD4^+^ T cell responses in 3 COVID-19 patients (1 HIV^–^/aTB^–^, 1 HIV^+^/aTB^–^, and 1 HIV^+^/aTB^+^). (**B**) Comparison of the prevalence and frequencies of SARS-CoV-2– and *M. tuberculosis*–specific CD4^+^ T cells in COVID-19 patients stratified by HIV or aTB coinfection. The proportion of responders to each pathogen (S: SARS-CoV-2 and M: *M. tuberculosis*) is presented with pies at the top of the graph. Statistical comparisons were performed using the χ^2^ test. Participants were grouped according to their HIV and/or TB status. Black bars represent the medians. (**C**) Comparisons of the frequencies of *M. tuberculosis*–specific CD4^+^ T cells in a cohort recruited before the emergence of COVID-19 (2018, *n* = 114), SARS-CoV-2–uninfected hospitalized controls (*n* = 29), and COVID-19 cases (*n* = 76). Participants were stratified according to their HIV and/or TB status. Statistical comparisons were defined using a Kruskal-Wallis test adjusted for multiple comparisons (Dunn’s test) for each subgroup. (**D**) Representative flow cytometry plots of HLA-DR expression on TNF-α–producing *M. tuberculosis*–specific CD4^+^ T cells in 3 COVID-19 patients (1 HIV^–^/aTB^–^, 1 HIV^+^/aTB^–^, and 1 HIV^+^/aTB^+^). (**E**) Summary graph of HLA-DR expression on *M. tuberculosis*–specific CD4^+^ T cells in a cohort recruited before the emergence of COVID-19 (2018), SARS-CoV-2–uninfected hospitalized controls, and COVID-19 cases stratified according to HIV and TB status. The phenotype of *M. tuberculosis*–specific CD4^+^ T cells was assessed only in those with response greater than 20 events.

To better understand the potential impact of COVID-19 on *M. tuberculosis* coinfection, we compared the frequency and phenotype of *M. tuberculosis*–specific CD4^+^ T cells in COVID-19 patients, hospitalized non–COVID-19 controls, and outpatient participants with latent TB (LTBI) or aTB recruited to unrelated studies prior to the emergence of the COVID-19 pandemic (Supplemental Table 3). *M. tuberculosis*–specific T cell responses were also assessed using a whole-blood assay (Figure 7A). Because HIV infection is known to decrease *M. tuberculosis*–specific CD4^+^ T cell response and aTB induces significant changes in the phenotype of *M. tuberculosis*–specific CD4^+^ T cells (46), patients were grouped according to their HIV and TB status for this analysis. The proportion of CD4 responders to SARS-CoV-2 and *M. tuberculosis* were comparable in HIV-uninfected COVID-19 patients (~90%). In HIV-infected patients with COVID-19, the proportion of an *M. tuberculosis*–specific CD4 response was significantly lower compared with that of SARS-CoV-2 (48% vs. 83%, respectively, *P* = 0.013). Conversely, in COVID-19 patients with aTB, SARS-CoV-2 responses were only detected in 40% of participants, whereas 14/15 (93%) exhibited an *M. tuberculosis*–specific CD4 response (Figure 7B). We did not find any relationship between the extent of CD4 lymphopenia and the absence of *M. tuberculosis*–specific responses (data not shown). Upon comparison of the frequency of *M. tuberculosis*–specific CD4^+^ T cells between the current cohort and the 2018 prepandemic cohort with LTBI, we found that the magnitude of *M. tuberculosis*–specific CD4^+^ T cells was approximately 5-fold lower in the HIV-uninfected COVID-19 group and approximately 2-fold lower in the HIV-infected COVID-19 group compared with prepandemic samples (medians: 0.17% vs. 0.53% for HIV^–^, *P* < 0.0001 and 0.09% vs. 0.17% for HIV^+^, *P* = 0.052, respectively). However, comparable frequencies were observed in those with aTB (medians: 0.35% for COVID-19 vs. 0.53% for prepandemic cohort, *P* = 0.3; Figure 7C). These data suggest that acute SARS-CoV-2 infection may diminish the pool of *M. tuberculosis*–specific memory T cell responses.

Last, HLA-DR expression on *M. tuberculosis*–specific CD4^+^ T cells has been shown to be a robust marker to distinguish active or subclinical TB from latent *M. tuberculosis* infection, regardless of HIV infection (46, 47). Thus, to define whether COVID-19 can promote *M. tuberculosis* reactivation, we compared the expression of HLA-DR on *M. tuberculosis*–specific CD4^+^ T cells in the different cohorts (Figure 7D). In participants without aTB, no difference in the expression of HLA-DR was observed between COVID-19 patients, hospitalized non–COVID-19 controls, and the 2018 prepandemic cohort, irrespective of their HIV status (Figure 7E). Moreover, in these patients, the memory maturation profile and expression of other activation markers (such as CD38, Ki67, and PD-1) in *M. tuberculosis*–specific CD4^+^ T cells were similar between COVID-19 patients and hospitalized non–COVID-19 controls (Supplemental Figure 4). In aTB patients, elevated HLA-DR expression was observed compared with latently infected individuals, as expected. However, although not statistically significant, the proportion of activated *M. tuberculosis*–specific CD4^+^ T cells tended to be higher in COVID-19–coinfected patients compared with the non–COVID-19 group (median: 74 %, IQR: 49%–94% vs. 57.7%, IQR: 50%–77%, respectively; Figure 7E). This suggests that acute COVID-19 does not promote the reactivation of latent *M. tuberculosis* infection but could enhance the activation of the *M. tuberculosis*–specific CD4^+^ T cell response during active TB.

## Discussion

In this study, using a cohort of acute COVID-19 cases and SARS-CoV-2–uninfected hospitalized patients, we interrogated the SARS-CoV-2–specific CD4^+^ T cell response patterns in relation to various measures of clinical disease severity to better understand the immune determinants of COVID-19 clinical course. Moreover, in a subset of patients, we investigated whether HIV and/or TB coinfections affected the CD4 response against SARS-CoV-2 and conversely, whether COVID-19 affected the *M. tuberculosis*–specific CD4 response.

First, by measuring the prevalence of SARS-CoV-2–specific CD4 responses, we showed that SARS-CoV-2–reactive CD4^+^ T cells were detected in a substantial proportion of SARS-CoV-2–uninfected patients (~34%). This is in accordance with several studies reporting that SARS-CoV-2 cross-reactive memory T cells are detectable in 20% to 50% of individuals with no prior exposure to SARS-CoV-2 (8, 10, 15–20). Limited information is available regarding the phenotype and function of these memory responses. Our data showed that SARS-CoV-2–responding CD4^+^ T cells were qualitatively different in acute COVID-19 cases compared with uninfected individuals. In the former group, SARS-CoV-2–specific CD4^+^ T cells almost exclusively displayed an early differentiated memory phenotype and limited capacity to produce IFN-γ; in the latter group, SARS-CoV-2–responsive CD4^+^ T cells preferentially exhibited a late differentiated memory phenotype and were enriched in GrB, suggesting that cytotoxic memory CD4^+^ T cells could be a relevant component in SARS-CoV-2 immunity, as previously described for other viral infections (48). However, to date, the functional role for preexisting cross-reactive T cell memory in COVID-19 remains unproven. In a comprehensive review, Leipsitch et al. describe 3 possible scenarios outlining potential mechanisms by which cross-reactive memory T cells could confer some form of protection against COVID-19 by reducing the viral burden and/or limiting disease severity or its duration (49).

Several publications have reported that severe COVID-19 elicits drastic changes in the overall distribution and phenotypical landscape of circulating T cells, characterized by severe lymphopenia (preferentially affecting CD8 T cells) and widespread T cell activation (4–6). Furthermore, an immune signature of the SARS-CoV-2–specific T cell response correlating with COVID-19 severity is also emerging (8–13). Most of these studies compared patients with very divergent forms of disease (hospitalized vs. nonhospitalized patients, convalescent patients who had mild or severe disease, or hospitalized vs. convalescent patients). Here, we report on the immune profile of SARS-CoV-2 CD4 response in hospitalized acute COVID-19 patients stratified by disease severity based on multiple clinical parameters of known relevance in COVID-19 outcome. Our data showed that the quality rather than the quantity of SARS-CoV-2–specific CD4^+^ T cells may contribute to an efficient COVID-19 immune response as previously described for other viral infections (50). Indeed, more severe forms of COVID-19 correlate with the SARS-CoV-2–specific CD4 response, displaying a limited capacity to produce IFN-γ, reduced expression of GrB and Ki-67, and elevated expression of HLA-DR. This is in agreement with other reports showing that reduced IFN-γ production characterizes severely ill patients (11, 12). Moreover, the overall profile of SARS-CoV-2–specific CD4^+^ T cells differed significantly between patients who survived COVID-19 and patients who died. The altered Th1 profile observed in severe COVID-19, reminiscent of an exhausted phenotype, could contribute to increased inflammation with poorer viral control. It thus remains to be seen whether recovery from COVID-19 can induce long-lasting, efficient memory T cells, regardless of the severity of the COVID-19 episode.

In this study, we also report the impact of HIV, TB, and HIV/TB coinfection on SARS-CoV-2 immunity. The clinical and epidemiological interactions of COVID-19 with TB and/or HIV-1 pose an additional health threat. In South Africa, 2 large epidemiological studies have shown that TB and HIV-1 were independently associated with increased risk of severe COVID-19 and death (27, 28). Although comorbidities associated with HIV-1 and TB may primarily drive COVID-19 severity in these populations, it is also plausible that HIV- and/or TB-associated immune dysregulation may contribute to heightened risk.

To date, the immunological impact of HIV on SARS-CoV-2 immune response has been mainly reported in isolated or limited cases of coinfection (51–53). Only 2 studies have measured the effect of HIV-1 infection on the overall profile of T cells in COVID-19 cases: Karim et al. showed that viremic HIV-infected COVID-19 patients exhibited lower frequencies of tissue-homing CXCR3^+^ CD8^+^ T cells and higher T cell activation compared with HIV-uninfected patients (54). Similarly, Sharov showed that viremic HIV-infected COVID-19 patients displayed enhanced exhaustion of their T cell compartment (55). This suggests that systemic immune activation associated with untreated HIV could skew the SARS-CoV-2 immune response. In our study, where most of the HIV^+^/aTB^–^ participants were virally suppressed (17 out of 22), we showed that HIV infection alone did not alter the functional and phenotypical profile of SARS-CoV-2 CD4^+^ T cells compared with HIV-uninfected patients. However, HIV-1–infected patients characteristically displayed a lower CD4^+^ T cell frequency compared with HIV-uninfected patients, which in turn was associated with lower magnitudes of SARS-CoV-2–specific CD4^+^ T cells and lower levels of IgG targeting SARS-CoV-2 nucleocapsid associated with total CD4^+^ T cell frequency. Moreover, in most HIV^+^/aTB^+^ patients with the most severe lymphopenia (CD4 frequency < 10%), SARS-CoV-2–specific responses were undetectable. These results suggest that preexisting lymphopenia, observed in untreated HIV-1 infection or in those with poor CD4 reconstitution despite ART usage, may impede the generation of T cell and/or antibody responses against SARS-CoV-2. These results will need to be confirmed in a larger cohort and could potentially be assessed by using different approaches (such as the agnostic activation-induced markers assay) to confirm the inability of lymphopenic patients to mount a T cell response to SARS-CoV-2. Despite COVID-19 and aTB coinfection cases reported in multiple countries (56–58), immunological data on the SARS-CoV-2 response in the context of active TB coinfection is scarce. Given that both diseases can elicit a hyperinflammatory state in the lung with overlap in the cytokine and chemokine profile found in broncho-alveolar lavage samples during severe COVID-19, TB, or HIV/TB coinfection (59, 60), it can be speculated that one disease may exacerbate the other, leading to unfavorable outcomes. One study recently showed, using an IFN-γ release assay, that aTB impairs the ability to mount a SARS-CoV-2–specific immune response in coinfected subjects (61). Here, we showed that active TB coinfection skewed the functional profile of SARS-CoV-2–specific CD4^+^ T cells, leading to a reduction of their polyfunctional capacity. It is possible that the excessive inflammation triggered by COVID-19 and TB coinfection underlies the premature functional exhaustion of SARS-CoV-2–specific T cells. Future studies to specifically examine differences in the inflammatory environment between COVID-19 and COVID-19/aTB patients in the blood and lung would shed more light on the interplay between the 2 diseases.

Additionally, in countries where the prevalence of latent *M. tuberculosis* infection is high, the profound lymphopenia induced by SARS-CoV-2 and use of steroids as a treatment for COVID-19 could (a) predispose patients to TB reactivation as a consequence of a transient suppression of cellular immunity and/or (b) increase the risk of progressive primary TB infection by reducing the pool of memory T cells targeting *M. tuberculosis*. We showed in this study that COVID-19 did not induce a concomitant activation of *M. tuberculosis*–specific CD4^+^ T cells, suggesting that acute SARS-CoV-2 infection may not immediately result in progression of latent *M. tuberculosis* to subclinical or active TB disease. However, we found a significant reduction in the frequency of *M. tuberculosis*–specific CD4^+^ T cells in COVID-19 patients compared with healthy prepandemic participants with LTBI. Because an intact T cell response is an essential component in *M. tuberculosis* control, this decline in *M. tuberculosis*–specific CD4^+^ T cells could affect the ability of the host to control latent or new *M. tuberculosis* infection. However, longitudinal studies are required to investigate whether T cell normalization after COVID-19 recovery is accompanied by homeostatic reexpansion or peripheral redistribution of the *M. tuberculosis*–specific memory T cell pool. Furthermore, it remains to be assessed whether alterations in the frequency or phenotype of SARS-CoV-2–specific CD4^+^ T cells, observed in the context of HIV and aTB coinfections, have an impact on COVID-19 clinical outcome, as the limited number of patients and the cross-sectional design of this study precluded speculation on this issue.

Overall, our results showed that the functional and phenotypical signature of SARS-CoV-2–specific CD4^+^ T cells, rather than magnitude, was associated with COVID-19 severity in hospitalized patients. These results further advance our knowledge of COVID-19 immunopathology, inform potential correlates of protection, and could provide a rationale for future evaluation of novel vaccine responses. Moreover, our findings revealed potential mechanisms by which HIV-1 and TB coinfections could exacerbate COVID-19 pathology.

## Methods

### Study cohorts

#### Hospitalized COVID-19 and non–COVID-19 patients.

We enrolled 133 hospitalized patients (95 with confirmed acute COVID-19 and 38 SARS-CoV-2 uninfected) from Groote Schuur Hospital in Cape Town, South Africa, between June and August 2020. The clinical characteristics of all patients included in this study are presented in Table 1, and the comparisons of the clinical characteristics between discharged and deceased COVID-19 patients are presented in Supplemental Table 2.

#### Case-control study (2018).

To compare the frequency and profile of *M. tuberculosis*–specific CD4^+^ T cells between samples collected before and during the SARS-CoV-2 pandemic, we used data generated from participants recruited at the Ubuntu Clinic, Site B, Khayelitsha, between March 2017 and December 2018. This cohort has been described in detail (62) and the clinical characteristics of the study participants are shown in Supplemental Table 3. Briefly, 122 adults (age 25 or older) were included in this study and classified into 4 groups according to their HIV-1 and TB status: LTBI/HIV^–^ (*n* = 24), LTBI/HIV^+^ (*n* = 30), aTB/HIV^–^ (*n* = 32), and aTB/HIV^+^ (*n* = 36). The median age was comparable between the 4 groups (median: 35 years, IQR: 31–45). All active TB cases were sputum Xpert *M. tuberculosis*/RIF (Cepheid) positive and had clinical symptoms and/or radiographic evidence of TB. The latent TB group were all asymptomatic, had a positive IFN-γ release assay (IGRA, QuantiFERON-TB Gold In-Tube), tested sputum Xpert *M. tuberculosis*/RIF negative, and exhibited no clinical evidence of active TB. HIV-infected participants with LTBI had a significantly lower plasma HIV-1 viral load and higher absolute CD4 count compared with the HIV-infected aTB group. These differences were due to higher ART usage in the LTBI group compared with the aTB group.

#### Convalescent COVID-19 donors.

Flow cytometry data were also available from a limited number of COVID-19 convalescent patients (*n* = 9). These participants were health care workers recruited between July and September 2020 from Groote Schuur Hospital in Cape Town. All had a SARS-CoV-2 PCR-positive test, had mild symptoms, and did not require hospitalization. All participants were symptom free at the time of sampling. Blood samples were obtained at a median of 4.7 weeks after the SARS-CoV-2 PCR test.

### Clinical data

At enrollment, participants’ clinical status was assessed according to the WHO ordinal scale based on their requirements for oxygen and supportive therapy (34). The WHO scale is the following: WHO 2 is ambulatory with limitation of activities, WHO 3 is hospitalized without requiring supplemental oxygen, WHO 4 is hospitalized with oxygen therapy by mask or nasal prongs, WHO 5 is hospitalized and requiring noninvasive ventilation or use of high-flow oxygen devices, WHO 6 is hospitalized and receiving invasive mechanical ventilation, and WHO 7 is hospitalized and receiving invasive mechanical ventilation and additional organ support such as extracorporeal membrane oxygenation. Absolute CD4 count (for HIV-infected patients) and WCC were obtained from patients’ medical files from the date closest to research blood collection. CRP, ferritin, D-dimer, LDH, and HIV-1 viral load were measured from blood collected at enrollment. All clinical tests were performed by the South African National Health Laboratory Services (NHLS). Posteroanterior chest radiographs were assessed for the total percentage of the lung fields unaffected by any visible pathology. Thus, in the COVID-19 group, this score quantified the percentage of normal lung that was not visibly affected by known features of COVID-19 pneumonia on the radiograph. In patients with TB or other respiratory infections, this score similarly quantified the percentage of normal lung not visibly affected by the relevant pathology on the radiograph. Individuals with a normal chest radiograph would thus score 100%.

### Measurement of SARS-CoV-2 nucleocapsid–specific IgG in plasma

SARS-CoV-2–specific antibodies were assayed by the Elecsys anti–SARS-CoV-2 immunoassay (Roche). This semiquantitative electrochemiluminescent immunoassay measures SARS-CoV-2 nucleocapsid–specific IgG. The assay was performed by the NHLS and interpreted according to manufacturer’s instructions (Roche, V 1.0 2020-05). Results are reported as numeric values in form of a cutoff index (signal sample/cutoff), where a cutoff index less than 1.0 corresponds to nonreactive plasma and a cutoff index of 1.0 or greater to reactive plasma. At 14 days after SARS-CoV-2 PCR confirmation, the sensitivity and specificity of the Elecsys anti–SARS-CoV-2 immunoassay is reported as 99.5% (95% CI, 97.0% to 100.0%) and 99.80% (95% CI, 99.69% to 99.88%), respectively (63–65).

### Whole blood–based T cell detection assay

Blood was collected in sodium heparin tubes and processed within 3 hours of collection. The whole-blood assay was adapted from the protocol described by Hanekom et al. (66). We adapted this assay to detect SARS-CoV-2–specific T cells using synthetic SARS-CoV-2 PepTivator peptides (Miltenyi Biotec), consisting of 15-mer sequences with 11 amino acids, overlap covering the immunodominant parts of the spike (S) protein, and the complete sequence of the nucleocapsid (N) and membrane (M) proteins (67). All peptides were combined in a single pool and used at a final concentration of 1 μg/mL. Briefly, 400 μL whole blood was stimulated with the SARS-CoV-2 S, N, and M protein peptide pool or a pool of 300 *M. tuberculosis*–derived peptides (Mtb300, 2 μg/mL, provided in-house; ref. 68) at 37°C for 5 hours in the presence of costimulatory antibodies against CD28 (clone 28.2) and CD49d (clone L25) (1 μg/mL each; BD Biosciences) and Brefeldin-A (10 μg/mL, MilliporeSigma). Unstimulated blood was incubated with costimulatory antibodies, Brefeldin-A, and an equimolar amount of DMSO. Red blood cell lysis and white cell fixation were performed in a single step using a transcription factor fixation buffer (eBioscience, Thermo Fisher Scientific) for 20 minutes. Cells were then cryopreserved in freezing media (50% FBS, 40% RPMI, and 10% dimethyl sulfoxide) and stored in liquid nitrogen until batched analysis.

### Cell staining and flow cytometry

Cell staining was performed on cryopreserved cells that were thawed, washed, and permeabilized with a transcription factor perm/wash buffer (eBioscience, Thermo Fisher Scientific). Cells were then stained at room temperature for 45 minutes with antibodies for CD3 BV650 (OKT3, BioLegend), CD4 BV785 (OKT4, BioLegend), CD8 BV510 (RPA-8, BioLegend), CD19-BV750 (HIB19, BioLegend), CD45RA Alexa Fluor 488 (HI100, BioLegend), CD27 PE-Cy5 (1A4CD27, Beckman Coulter), CD38 APC (HIT2, BD Biosciences), HLA-DR BV605 (L243, BioLegend), Ki67 PerCP-Cy5.5 (B56, BD Biosciences), PD-1 PE (J105, eBioscience, Thermo Fisher Scientific), GrB BV421 (BG11, BD Biosciences), IFN-γ BV711 (4S.B3, BioLegend), TNF-α PE-Cy7 (MAB11, BioLegend), and IL-2 PE/Dazzle 594 (MQ1-17H12, BioLegend). Samples were acquired on a BD Biosciences LSR II and analyzed using FlowJo v9.9.6. A positive response was defined as any cytokine response that was at least twice the background of unstimulated cells. To define the phenotype of SARS-CoV-2–specific CD4^+^ T cells, a cutoff of 20 events was used.

### Statistics

Graphical representations were performed in Prism (v9; GraphPad Software) and JMP (v14.0.0; SAS Institute). Statistical tests were performed in Prism. Nonparametric tests were used for all comparisons. The Kruskal-Wallis test with Dunn’s multiple-comparison test was used for group comparisons, and the Mann-Whitney and Wilcoxon’s matched-pair test were used for unmatched and paired samples, respectively. *P* values less than 0.05 were considered to indicate statistical significance.

### Study approval

This study was approved by the University of Cape Town’s Faculty of Health Sciences Human Research Ethics Committee (207/2020 and 050/2015), and written informed consent was obtained from all participants with the capacity to provide it. Relatives provided proxy consent for participants without capacity to consent for themselves (e.g., because of decreased level of consciousness). In cases where participants regained capacity, informed consent was obtained from them directly at that time.

## Author contributions

CR, EDB, CS, RD, and RJW designed the study. EDB, CS, RD, and RTG recruited the study participants. RJW and SW facilitated clinical recruitment. EDB and CR performed the flow experiments. CR performed the data analysis and interpretation. FA supervised sample collection and processing. BWA conceptualized the radiographic reading method. QSH interpreted the radiological data. MH supervised the serology test. KAW, CSLA, and AS provided critical reagents. CR wrote the manuscript with all authors contributing by providing critical feedback.

## Supplementary Material

Supplemental data

## Figures and Tables

**Table 1:**
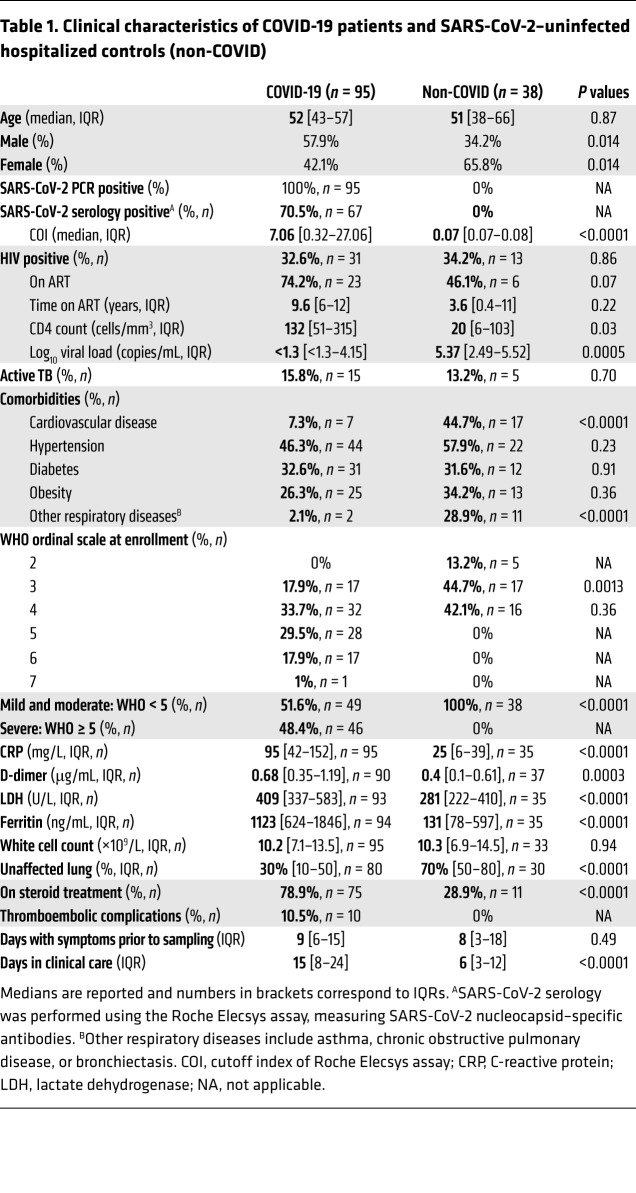
Clinical characteristics of COVID-19 patients and SARS-CoV-2–uninfected hospitalized controls (non-COVID)

## Version 1 05/04/2021

In-Press Preview

## Version 2 06/15/2021

Electronic publication
