# Supplementary material for: Relationship of SARS-CoV-2-specific CD4 response to COVID-19 severity and impact of HIV-1 and Tuberculosis co-infection
Source: J Clin Invest. Author manuscript; Available in PMC 2021 Jun 23. (PMC8203446; doi:10.1172/JCI149125)
Supplement: Supplementary material [file EMS124717-supplement-Supplementary_material.pdf]

## Supplemental Table 1

**COVID-19 patients**

| **Groups** | **HIV+/aTB-** | **HIV+/aTB+** |
|---|---|---|
| **N** | **23** | **8** |
| **Age** (median, IQR) | **47** [42-53] | **40** [33-54] |
| **Male** (%) | **43.5%** | **37.5%** |
| **On ART** (%, n) | **78.3%,** n=18 | **62.5 %***, n=5 |
| **CD4 count** (cells/mm$^3$, IQR) | | |
| On ART | **264** [134-359] | **80** [32-184] |
| ART-naïve | **40** [8-120] | **106** [17-106] |
| **Log$_{10}$ Viral load** (median, IQR) | | |
| On ART | **<1.3** [<1.3-<1.3] | **3.5** [1.9-5] |
| ART-naïve | **4.4** [2.7-4.7] | **4.7** [4.2-5.3] |

**Supp Table 1. Clinical characteristics of HIV-infected and HIV/aTB co-infected COVID-19 participants.** *: Despite reporting anti-retroviral treatment (ART) usage, it is most likely that most of the HIV+/aTB+ patients have defaulted treatment, with only one out of eight patients exhibiting an undetectable HIV-1 viral load with a CD4 count of 209 cells/mm$^3$.

## Supplemental Table 2

| | **SURVIVED** **70.5%** (n=67) | **DECEASED** **29.5%** (n=28) | ***P-values*** |
|---|---|---|---|
| **Age** (median, IQR) | **50** [42-56] | **55** [44-65] | **0.029** |
| **Male** (%, n) | **50.7%** (n=34) | **75%** (n=21) | **0.029** |
| **HIV positive** (%, n) | **37.3%** (n=25) | **21.4%** (n=6) | 0.13 |
| on ART | **72%** (n=18) | **83.3%** (n=5) | 0.39 |
| Time on ART (years, IQR) | **9.5** [6-12] | **10** [3.5-11] | 0.75 |
| CD4 count (cells/mm$^3$, IQR) | **144** [53-332] | **113** [45-270] | 0.71 |
| Log Viral load | **<1.3** [<1.3-3.75] | **2.38** [<1.3-4.91] | 0.42 |
| **Active TB** (%, n) | **13.4%** (n=9) | **21.4%** (n=6) | 0.33 |
| **Co-morbidities** (%, n) | | | |
| Cardiovascular | **7.5%** (n=5) | **7.1%** (n=2) | 0.96 |
| Hypertension | **43.3%** (n=29) | **53.6%** (n=15) | 0.36 |
| Diabetes | **34.3%** (n=23) | **46.4%** (n=13) | 0.25 |
| Obesity | **29.8%** (n=20) | **35.7%** (n=10) | 0.57 |
| Other respiratory diseases | **10.4%** (n=7) | **-** | **-** |
| **SARS-CoV2 serology positive*** (%, n) | **67.2%** (n=45) | **75%** (n=21) | 0.45 |
| COI (median, IQR) | **5.5** [0.25-23.3] | **15.5** [0.73-39.3] | 0.057 |
| **WHO scale at enrolment** (%, n) | | | |
| 3 | **25.4%** (n=17) | **-** | - |
| 4 | **43.3%** (n=29) | **10.7%** (n=3) | **0.002** |
| 5 | **22.4%** (n=15) | **46.4%** (n=13) | **0.019** |
| 6 | **8.9 %** (n=6) | **39.2%** (n=11) | **0.0004** |
| 7 | **-** | **3.6%** (n=1) | - |
| **Severe: WHO ≥5** (%, n) | **31.3%** (n=21) | **89.3%** (n=25) | **<0.0001** |
| **Cycle threshold value SARS PCR**** (IQR) | **30.6** [24.8-33.9] | **29.1** [25.3-34.2] | 0.83 |
| **CRP** (mg/L, IQR, n) | **66** [31-136] (n=67) | **129** [58-222] (n=28) | **0.015** |
| **D-dimer** (µg/mL, IQR, n) | **0.54** [0.32-0.98] (n=63) | **0.85** [0.59-2.0] (n=27) | **0.015** |
| **LDH** (U/L) | **385** [313-513] (n=66) | **543** [387-689] (n=27) | **0.0009** |
| **Ferritin** (ng/mL, IQR, n) | **921** [512-1581] (n=96) | **1719** [1031-2279] (n=28) | **0.002** |
| **White cell count** (x10$^9$/L, IQR, n) | **8.9** [6.3-12.2] (n=67) | **11.9** [9.0-16.6] (n=28) | **0.005** |
| **Unaffected lung** (%, IQR, n) | **40%** [20-70] (n=59) | **20%** [0-40] (n=27) | **<0.0001** |
| **On steroid treatment** (%, n) | **73.1%** (n=49) | **92.9%** (n=26) | **0.032** |
| **Days in hospital** (%, IQR) | **11** [6-23] | **15** [7-22] | 0.54 |

**Supp Table 2. Clinical characteristics of COVID-19 patients who survived or died.**
*: SARS-CoV-2 serology was performed using the Roche Elecsys assay, measuring SARS-CoV-2 nucleocapsid-specific antibodies. **: SARS-CoV-2 polymerase chain reaction (PCR) was performed using the Allplex™ 2019-nCoV Assay manufactured by Seegene.
Medians are reported and numbers in brackets correspond to interquartile range [IQR]. ART: antiretroviral treatment, COI: Cut-off index of Roche Elecsys assay, CRP: C-Reactive protein, LDH: lactate dehydrogenase.

## Supplemental Table 3

| Groups | HIV-/aTB- | HIV+/aTB- | HIV-/aTB+ | HIV+/aTB+ |
|---|---|---|---|---|
| **N** | **24** | **30** | **32** | **36** |
| **Age** (median, IQR) | **33** [28-43] | **34** [32-41] | **34** [32-42] | **37** [32-45] |
| **Male** (%) | **58.3%** | **23.3%** | **50%** | **66.7%** |
| **CD4 count** (cells/$mm^3$, IQR) | nd | **481** [358-700] | nd | **268** [141 - 400] |
| **$Log_{10}$ Viral load** (median, IQR) | na | **<1.3** [<1.3-4.18] | na | **4.59** [2.19-5.00] |
| **On ART** (%) | na | **80.6 %** | na | **38.9%** |

**Supp Table 3. Clinical characteristics of 2018 case-control cohort.**
ART: anti-retroviral treatment. nd: not done, na: not applicable.

## Supplemental Figure 1

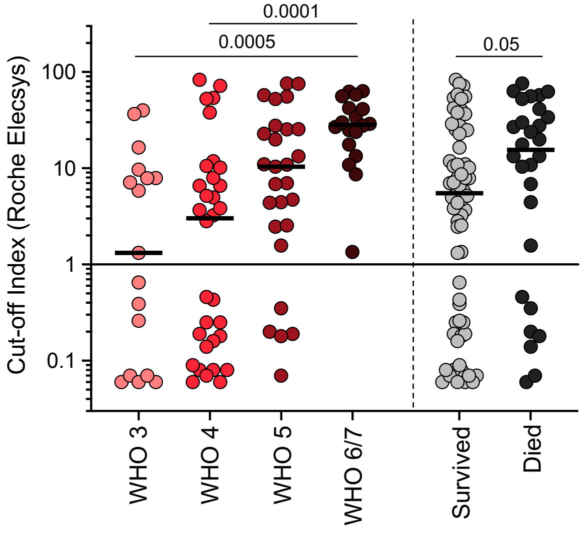


**Supp Figure 1**. **Magnitude of SARS-CoV-2-specific serological response** (defined using the Roche Elecsys® assay) in COVID-19 patients (n=94) stratified according to WHO ordinal score and outcome. Statistical comparisons were defined using a Kruskal-Wallis test, adjusted for multiple comparisons (Dunn's test) for the different WHO groups and the Mann-Whitney test to compare COVID-19 patients who survived or died. The plain horizontal line depicts the positivity cut-off as defined by manufacturer.

## Supplemental Figure 2

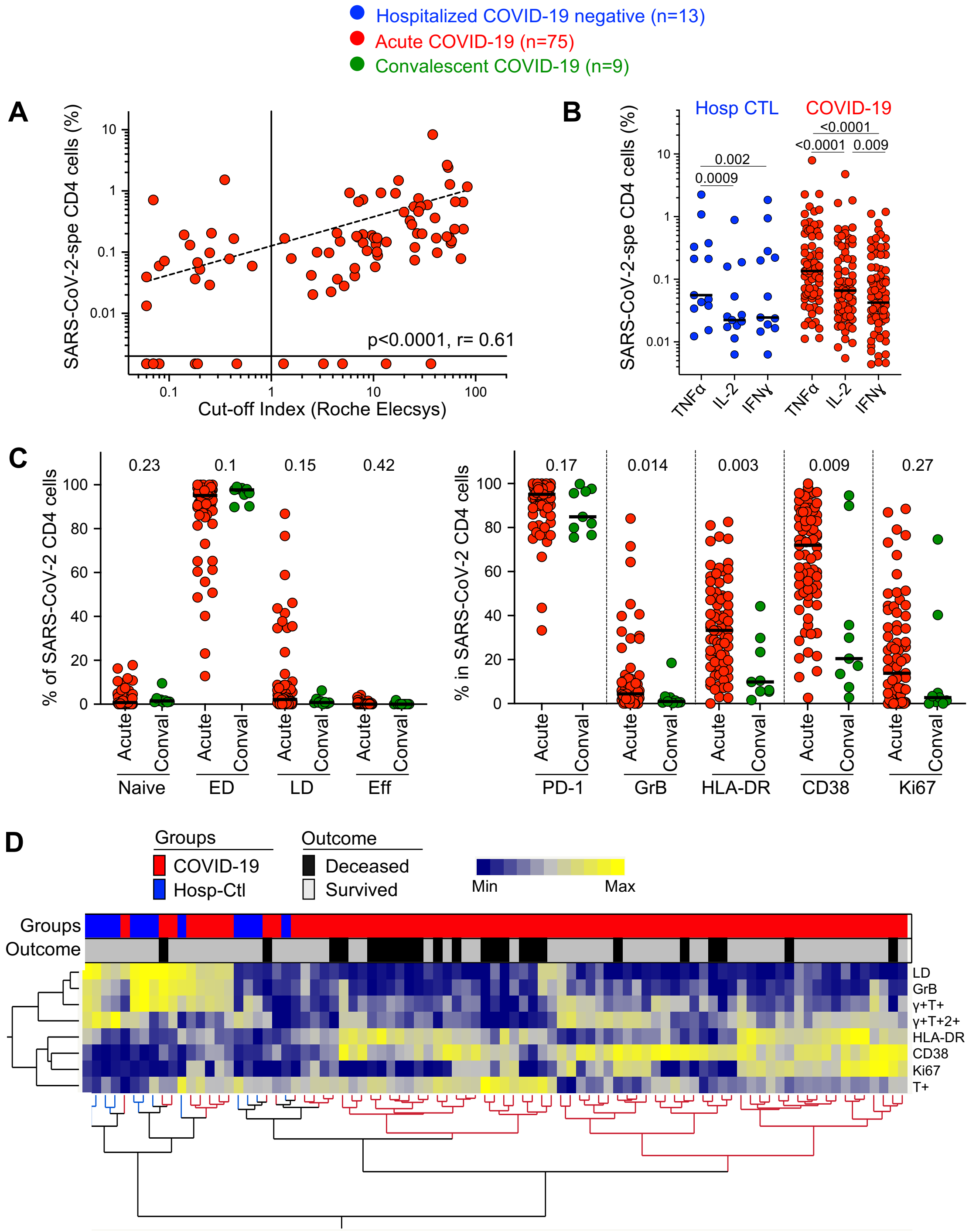


**Supp Figure 2**. **(A)** Association between the magnitude of SARS-CoV-2-specific serological response (defined using the Roche Elecsys® assay) and the frequency of SARS-CoV-2 CD4 T cells in COVID-19 patients. Correlation was tested by a two-tailed non-parametric Spearman rank test. **(B)** Comparison of frequency of IFNγ-, TNFα-, and IL2- in producing SARS-CoV-2-reactive CD4 T cells in acute COVID-19 cases (red) and hospitalized SARS-CoV-2-uninfected patients (blue). Only SARS-CoV-2 responders are depicted. **(C)** Comparison of the memory (left) and activation (right) profile of SARS-CoV-2-specific CD4 T cells between acute COVID-19 cases (red) and convalescent patients (green). Statistical comparisons were calculated using the non-parametric Mann-Whitney test. **(D)** Non-supervised two-way hierarchical cluster analysis (Ward method) of eight functional or phenotypic attributes of SARS-CoV-2-specific CD4 T cells (the proportion of IFNγ+TNFα+IL2+, IFNγ-TNFα+IL2+ IFNγ-TNFα+IL2- and cells, the proportion of ED, and GrB, HLA-DR, CD38 and Ki67 expression). COVID-19 status and outcome for each patient is indicated at the top of the dendrogram.

## Supplemental Figure 3

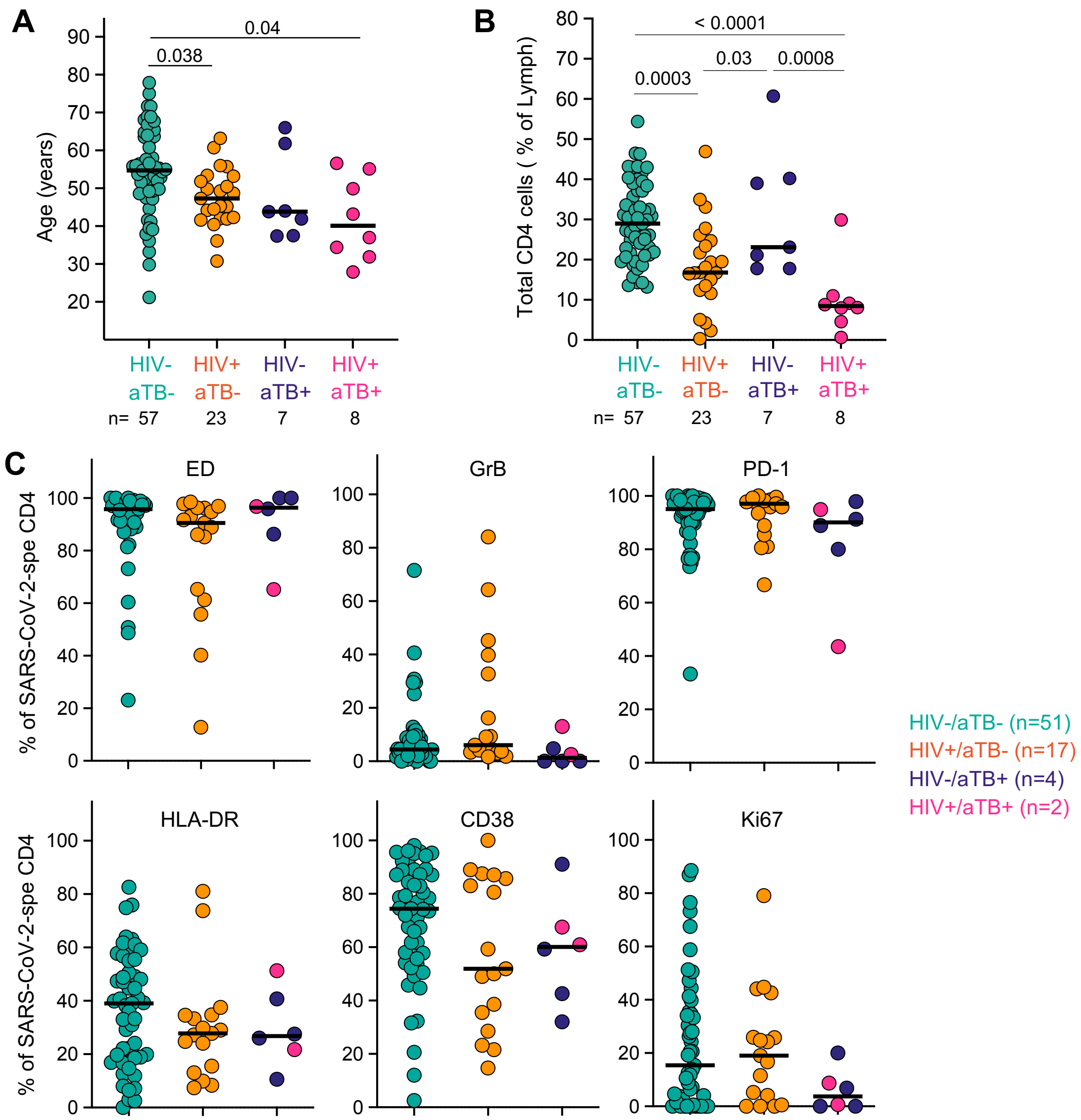


**Supp Figure 3. Clinical characteristics and phenotype of SARS-CoV-2-specific CD4 T cells in COVID-19 patients, grouped according to their HIV and TB status. (A)** Comparison of the age of COVID-19 patients based on their HIV and TB status. **(B)** Comparison of the frequency of total CD4 T cells based on patient's' HIV and TB status. Statistical comparisons were performed using a Kruskal-Wallis test, adjusted for multiple comparisons (Dunn's test). **(C)** Memory and activation profile of SARS-CoV-2-specific CD4 T cells in COVID-19 cases, stratified by HIV and/or aTB co-infection. ED: Early differentiated (CD45RA-CD27+). The phenotype of SARS-CoV-2-specific CD4 T cells was assessed only on response with at least 20 events.

## Supplemental Figure 4

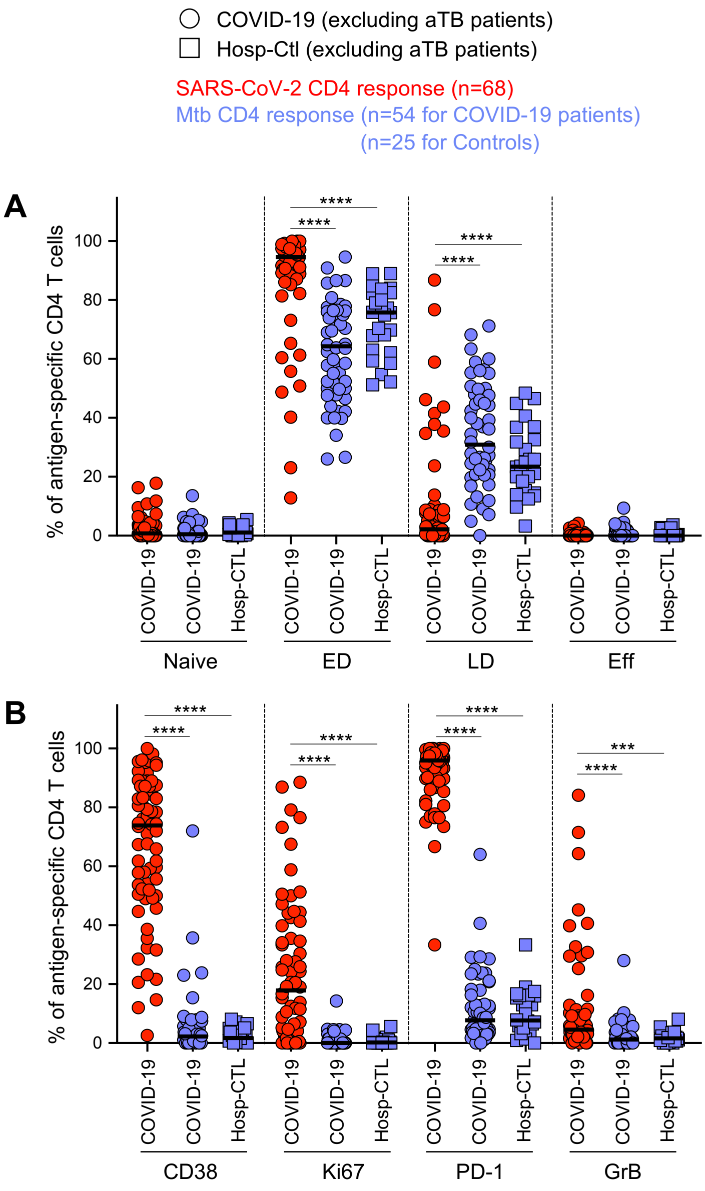


**Supp Figure 4. Comparison of the memory differentiation (A) and activation (B) profile of SARS-CoV-2- and Mtb-specific CD4 T cells in hospitalized COVID-19 and non-COVID-19 patients**. Statistical comparisons were performed using a Kruskal-Wallis test, adjusted for multiple comparisons (Dunn's test). ****: $p < 0.0001$, ***: $p = 0.0001$. Naïve (CD45RA+CD27+), ED: early differentiated (CD45RA-CD27+), LD: late differentiated (CD45RA-CD27-), Eff: Effector (CD45RA+CD27-).

**Complete list of HIATUS consortium members (alphabetical order):**

1. Fatimah Abrahams, University of Cape Town
2. Saalikha Aziz, University of Cape Town
3. Nonzwakazi Bangani, University of Cape Town
4. John Black, Livingstone Hospital, Port Elizabeth
5. Melissa Blumenthal, University of Cape Town
6. Marise Bremer, University of Cape Town
7. Wendy Burgers, University of Cape Town
8. Maddalena Cerrone, University of Cape Town, Imperial College London and Francis Crick Institute
9. Zandile Ciko, University of Cape Town
10. Anna K Coussens, University of Cape Town and Walter and Eliza Hall Institute of Medical Research, and University of Melbourne
11. Remy Daroowala, Imperial College London and University of Cape Town
12. Angharad G Davis, Francis Crick Institute, University of Cape Town and University College London
13. Jantina de Vries, University of Cape Town
14. Elsa du Bruyn, University of Cape Town
15. Hanif G Esmail, University College London and University of Cape Town
16. Rene T Goliath, University of Cape Town
17. Siamon Gordon, University of Oxford
18. Yolande XR Harley, University of Cape Town
19. Amanda Jackson, University of Cape Town
20. Rachel P-J Lai, Imperial College London and Francis Crick Institute, London
21. Francisco Lakay, University of Cape Town
22. Fernando-Oneissi Martinez-Estrada, University of Surrey
23. Graeme Meintjes, University of Cape Town
24. Marc S Mendelson, University of Cape Town
25. Ntobeko Ntusi, University of Cape Town
26. Tari Papavarnavas, University of Cape Town
27. Alize Proust, Francis Crick Institute, London
28. Catherine Riou, University of Cape Town
29. Sheena Ruzive, University of Cape Town
30. Qonita Said-Hartley, University of Cape Town
31. Georgia Schafer, International Centre for Genetic Engineering and Biotechnology, Cape Town
32. Keboile Serole, University of Cape Town
33. Cari Stek, Imperial College London and University of Cape Town
34. Nicki Tiffin, University of Cape Town
35. Sean Wasserman, University of Cape Town
36. Claire Whitaker, University of Cape Town
37. Katalin A Wilkinson, Francis Crick Institute and University of Cape Town
38. Robert J Wilkinson, University of Cape Town, Imperial College London and Francis Crick Institute
39. Kennedy Zvinairo, University of Cape Town
